# Real-time pandemic surveillance using hospital admissions and mobility data

**DOI:** 10.1073/pnas.2111870119

**Published:** 2022-02-01

**Authors:** Spencer J. Fox, Michael Lachmann, Mauricio Tec, Remy Pasco, Spencer Woody, Zhanwei Du, Xutong Wang, Tanvi A. Ingle, Emily Javan, Maytal Dahan, Kelly Gaither, Mark E. Escott, Stephen I. Adler, S. Claiborne Johnston, James G. Scott, Lauren Ancel Meyers

**Affiliations:** ^a^Department of Integrative Biology, The University of Texas at Austin, Austin, TX 78712;; ^b^Santa Fe Institute, Santa Fe, NM, 87501;; ^c^Department of Statistics and Data Science, The University of Texas at Austin, Austin, TX 78712;; ^d^Department of Operations Research and Industrial Engineering, The University of Texas at Austin, Austin, TX 78712;; ^e^School of Public Health, The University of Hong Kong, Hong Kong, China;; ^f^Texas Advanced Computing Center, The University of Texas at Austin, Austin, TX 78712;; ^g^Department of Women’s Health, Dell Medical School, Austin, TX 78712;; ^h^Office of the Chief Medical Officer, City of Austin, Austin, TX 78721;; ^i^Office of the Mayor, City of Austin, Austin, TX 78701;; ^j^Department of Neurology, Dell Medical School, The University of Texas at Austin, Austin, TX 78712;; ^k^Department of Information, Risk, and Operations Management, The University of Texas at Austin, Austin, TX 78712

**Keywords:** COVID-19, forecasting, healthcare usage, epidemiological data

## Abstract

Forecasting the burden of COVID-19 has been impeded by limitations in data, with case reporting biased by testing practices, death counts lagging far behind infections, and hospital census reflecting time-varying patient access, admission criteria, and demographics. Here, we show that hospital admissions coupled with mobility data can reliably predict severe acute respiratory syndrome coronavirus 2 (SARS-CoV-2) transmission rates and healthcare demand. Using a forecasting model that has guided mitigation policies in Austin, TX, we estimate that the local reproduction number had an initial 7-d average of 5.8 (95% credible interval [CrI]: 3.6 to 7.9) and reached a low of 0.65 (95% CrI: 0.52 to 0.77) after the summer 2020 surge. Estimated case detection rates ranged from 17.2% (95% CrI: 11.8 to 22.1%) at the outset to a high of 70% (95% CrI: 64 to 80%) in January 2021, and infection prevalence remained above 0.1% between April 2020 and March 1, 2021, peaking at 0.8% (0.7-0.9%) in early January 2021. As precautionary behaviors increased safety in public spaces, the relationship between mobility and transmission weakened. We estimate that mobility-associated transmission was 62% (95% CrI: 52 to 68%) lower in February 2021 compared to March 2020. In a retrospective comparison, the 95% CrIs of our 1, 2, and 3 wk ahead forecasts contained 93.6%, 89.9%, and 87.7% of reported data, respectively. Developed by a task force including scientists, public health officials, policy makers, and hospital executives, this model can reliably project COVID-19 healthcare needs in US cities.

## Significance

Forecasting COVID-19 healthcare demand has been hindered by poor data throughout the pandemic. We introduce a robust model for predicting COVID-19 transmission and hospitalizations based on COVID-19 hospital admissions and cell phone mobility data. This approach was developed by a municipal COVID-19 task force in Austin, TX, which includes civic leaders, public health officials, healthcare executives, and scientists. The model was incorporated into a dashboard providing daily healthcare forecasts that have raised public awareness, guided the city’s staged alert system to prevent unmanageable ICU surges, and triggered the launch of an alternative care site to accommodate hospital overflow.

As the COVID-19 pandemic emerged in the United States in early 2020, policy makers were forced to make decisions with limited information about the natural history, local prevalence, and transmission of the causative virus (severe acute respiratory syndrome coronavirus 2 [SARS-CoV-2]). Public health agencies and research institutions rapidly developed dashboards to track and forecast COVID-19 cases, hospitalizations, and mortality at multiple spatial scales using myriad data sources (1–8). Early policy responses referenced publicly available state or national COVID-19 mortality projections (1, 9, 10). As the course of the pandemic diverged across the United States, decision makers increasingly tracked and responded to trends in their own communities (4, 11–15).

Public demand for COVID-19 forecasts and the availability of new forms of data spurred rapid advances in pandemic modeling. The Institute for Health Metrics and Evaluation (IHME) launched one of the earliest and most widely cited COVID-19 forecasting dashboards, on March 26, 2020 (16). The White House Coronavirus Task Force first referenced IHME projections on March 29, 2020, when recommending a month-long extension of shelter-in-place orders (1, 6, 17). Data companies like SafeGraph and Cuebiq have made large volumes of granular mobility data freely available to the research community for characterizing changing behavioral patterns and their impacts on SARS-CoV-2 transmission (18–20). Johns Hopkins University and *The New York Times* have led the charge in publicly tracking key data throughout the pandemic (8, 21). As of June 2021, voluntarily maintained websites provide daily access to county-level case and mortality counts (8, 21), state hospital census and testing counts (3), epidemiological behavioral surveys (22), genomic data (23), anonymized case information (24), and government policies and responses (25). Since December 2020, the US Department of Health and Human Services and Centers for Disease Control and Prevention (CDC) have provided facility-level healthcare usage statistics (26).

The quality of COVID-19 data has varied through time and across populations as testing, healthcare, and reporting practices have shifted. Accounting for biases in our observational processes is critical to providing reliable situational awareness, investigating pandemic drivers and risks, and accurate forecasting. Case counts and test positivity can indicate changing risks, but are often biased by geographic and temporal variation in testing effort and priorities (27–30). For example, when COVID-19 antigen tests were initially distributed for proactive screening in schools and long-term care facilities, some states reported the combined antigen and PCR test results, while others did not (31). While COVID-19 mortality counts are likely underreported (32), they are a high priority outcome of interest for national forecasting efforts (33) and may provide the most accurate but substantially delayed signal of past transmission (34, 35). Often, case and mortality counts are analyzed jointly to reduce both delays and biases (4, 34, 36). COVID-19 healthcare data including hospital admissions, census, and ICU usage offer the fidelity of mortality data with a shorter lag, while also providing an immediate indication of healthcare resource needs. For example, COVID-19 hospitalizations have been used to estimate the impact of nonpharmaceutical interventions (37), provide healthcare demand forecasts (38–41), and guide mitigation policies (42, 43). However, such data can be biased by shifting demographics of COVID-19 patients, changes in admission criteria during surges, and the availability of post–acute care facilities (44, 45).

The municipal COVID-19 task force in the City of Austin, TX, developed a COVID-19 healthcare forecasting model that has guided regional pandemic responses since April 2020. The model is designed to provide robust, accessible, and holistic information about the changing pandemic situation. Using comprehensive COVID-19 hospital admissions and discharge data as well as cell phone GPS traces, the model estimates the impact of past policies and community behavior, real-time prevalence and transmission risks, and future COVID-19 hospitalizations and ICU needs. Here, we motivate our use of hospital admissions data by comparing the timeliness and fidelity of alternative indicators and then apply the model to characterize the first year of the COVID-19 pandemic in terms of the daily SARS-CoV-2 prevalence, transmission rate, case detection rate, and correlation between mobility and transmission. We then examine the impact of key policy and behavior shifts on these trends and retrospectively assess the performance of our 3-wk-ahead COVID-19 healthcare forecasts. These analyses led to two public dashboards, one tracking daily COVID-19 admissions from all area hospitals (46) and another providing COVID-19 healthcare forecasts (47). Both have been maintained since the spring of 2020 and continue to guide risk awareness, mitigation policies, and healthcare resource allocations in the fastest-growing large city in the United States, with a metropolitan area population approaching 2.3 million.

## Results

**Fig. 1.**
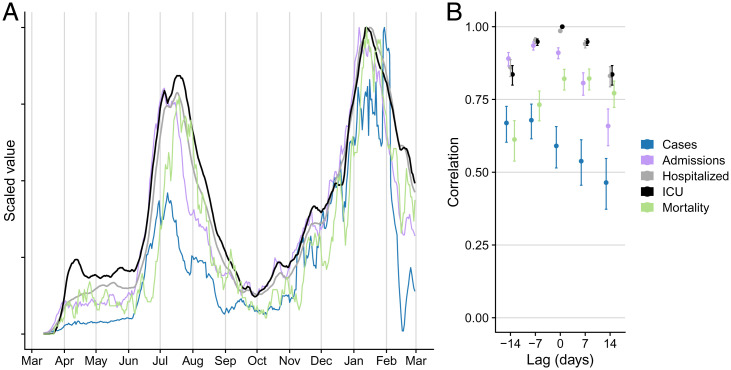
Fidelity and timeliness of COVID-19 data sources in the Austin, TX, MSA. (*A*) Scaled 7-d rolling averages of confirmed COVID-19 cases (8), COVID-19 hospital admissions, COVID-19 hospital census, COVID-19 ICU census, and COVID-19 mortality. Time series are scaled from zero to one. (*B*) Time-lagged correlations between all candidate predictors and ICU census at five lag intervals (–14, –7, 0, 7, and 14 d). Error bars indicate the 95% CI of the correlation coefficient for the specified lag. Negative *x*-axis lag values mean that the predictor leads the target (desirable); positive values mean the predictor lags the target.

A visual comparison of COVID-19 case counts, hospital admissions, hospital census, ICU census, and death counts in the Austin–Round Rock metropolitan statistical area (MSA) from March 13, 2020 through February 28, 2021 reveals persistent lags and different degrees of variability (Fig. 1*A*). Deaths tend to lag the other variables by several weeks; the three healthcare variables—-hospital admissions, hospital census (which includes general and ICU patients), and ICU census—-are smoother than case counts, with multiweek hospital stays causing the hospital census and ICU census to decline more slowly following peaks. Assuming that the goal of surveillance is to anticipate COVID-19 healthcare demand, we evaluate all variables in terms of the timing and strength of their correlation with COVID-19 healthcare usage indicators such as hospital and ICU census (Fig. 1*B*). Case counts and hospital admissions are strong leading indicators of hospital census, providing maximum correlations of 0.72 (95% CI: 0.66 to 0.77) 8 d ahead and 0.95 (95% CI: 0.94 to 0.96) 6 d ahead, respectively. They also strongly predict ICU census, with case counts achieving a maximum correlation of 0.68 (95% CI: 0.62 to 0.74) 5 d ahead and hospital admissions reaching 0.94 (95% CI: 0.92 to 0.95) 6 d ahead. Day-to-day noise in surveillance data can amplify uncertainty in estimating and communicating risk. Based on a 1-d lag autocorrelation, we expect hospital admissions (91%; 95% CI: 89 to 93%) to provide a smoother signal of changing risks than cases (58%; 95% CI: 50 to 64%) or deaths (75%; 95% CI: 70 to 79%).

**Fig. 2.**
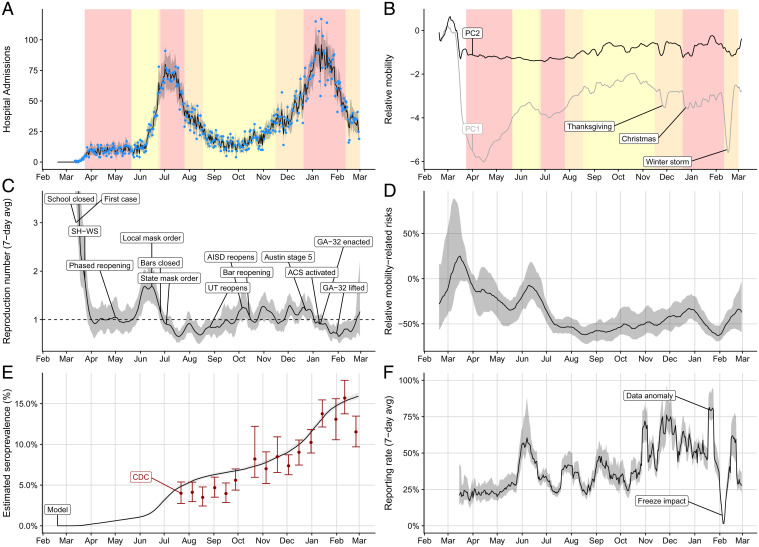
Estimated COVID-19 pandemic healthcare, mobility, and epidemiological trends in the Austin–Round Rock, TX, MSA from February 18, 2020 to February 28, 2021. (*A*) Median fitted (line) and observed (points) daily COVID-19 hospital admissions, with gray ribbon indicating the 95% prediction interval. (*B*) First two principal components derived from eight cell phone mobility variables provided by SafeGraph (19). Yellow, orange, and red shading in the top graphs indicate the timing of COVID-19 alert stages 3, 4, and 5, respectively, in the Austin MSA (46). (*C*) Estimated 7-d average reproduction number (*R_t_*) with gray 95% credible band. (*SI Appendix*, Fig. S4 shows the full range of values early in the pandemic.) Text boxes mark key policy changes and epidemiological events (details in *SI Appendix*, Table S7), with the following abbreviations: SH-WS indicates the March 24, 2020 Stay Home–Work Safe order; UT indicates the University of Texas at Austin; AISD indicates Austin’s largest public school system, Austin Independent School District; ACS indicates the Alternative Care Site established in a convention center to expand healthcare capacity; and GA-32 indicates a Texas order restricting elective surgeries, bars, and restaurants according to COVID-19 healthcare usage. (*D*) Transmission rates relative to baseline behavior from February 19, 2020 to March 1, 2021. Our model continually estimates this relationship between mobility and transmission, since increases and decreases in precautionary behavior can change this relationship. The graph compares the estimated transmission rate at each point in time to a hypothetical transmission rate that assumes no behavioral changes (i.e., the relationship between mobility and transmission remains fixed at a value estimated prior to wide adoption of COVID-19 safety measures). A positive (negative) value indicates that the observed transmission rate was higher (lower) than would be expected if precautionary behavior had remained constant. Shading indicates 95% credible bands. (*E*) Comparison between our projections for SARS-CoV-2 seroprevalence (black line with gray 95% credible bands) and estimates from a Texas-wide seroprevalence survey scaled to Austin (red points with 95% CIs) (48). (*F*) Estimated weekly case reporting rate, with gray 95% credible band. Values correspond to the proportion of cases infected on the given day (*x* axis) that are eventually reported. We indicate a data anomaly, in which thousands of backlogged cases were reported on a single day, and the impact of a catastrophic winter freeze that disrupted citywide testing and reporting operations (49–51).

Given the advantages of COVID-19 hospital admission data over the alternative indicators, we propose a forecasting model that uses admissions counts in combination with cell phone GPS data to estimate local transmission rates and project imminent healthcare surges (*SI Appendix*, Fig. S1). Specifically, we use particle filtering to fit an age- and risk-structured susceptible–exposed–infected–recovered (SEIR) model to daily reported COVID-19 hospital admissions, discharges, and in-hospital deaths (*Materials and Methods*). To capture unmeasured changes in exposure rates stemming from changes in policy and behavior, we assume that transmission rates depend on population mobility and simultaneously estimate time-dependent regression coefficients governing that relationship (Fig. 2).

The model yields COVID-19 hospital admissions estimates that mirror the observed data in the Austin MSA from March 13, 2020 through February 28, 2021 (Fig. 2*A*). We observe similar fidelity with respect to COVID-19 hospital census, ICU usage, discharge, and in-hospital mortality during the same time period (*SI Appendix*, Fig. S3). We estimate that the pandemic emerged in Austin on February 19, 2020 (interquartile range [IQR]: February 13–25), with the effective reproduction number (*R_t_*) reaching a maximum 7-d average of 5.8 (95% CrI credible interval [CrI]: 3.6 to 7.9) on March 5 (*SI Appendix*, Fig. S4). Following the citywide closure of schools on March 13 and Stay Home–Work Safe order on March 24, 2020 (52, 53), the estimated reproduction number dropped to a temporary low of 0.91 (95% CrI: 0.65 to 1.3) on April 6 (Fig. 2*C*). Although the reproduction number remained relatively flat through late April, the upper bound of the 95% CrI never fell below one. Following the White House’s Opening Up America Again guidelines, Texas reopened in phases starting May 1, 2020 (54–56). Within weeks, the estimated SARS-CoV-2 transmission began to increase, reaching a peak of 1.7 (95% CrI: 1.3 to 2.0) on June 6. To curb rising hospitalizations, the City of Austin enacted a mask order and limited gathering sizes on June 15 (57). Statewide, Texas closed bars on June 26 and enacted mask orders and gathering limits on July 3 (58, 59). The pandemic then slowed rapidly to the minimum detected *R_t_* of 0.65 (95% CrI: 0.52 to 0.77) on July 19. Between mid-August and mid-October, the University of Texas opened, with an estimated 30,000 students in Austin participating in hybrid instruction (60); Austin Independent School District, with an enrollment of over 80,000 students, returned to optional in-person instruction (61); and bars were reopened statewide (62). During this period, the reproduction number steadily increased to a high of 1.3 (95% CrI: 1.0 to 1.5) on October 31 and likely remained at or above 1.0 until January 18, 2021, producing an alarming winter surge.

Since May 2020, the city has maintained a public-facing dashboard (46) that tracks the 7-d moving average of COVID-19 hospital admissions and provides clear threshold values for activating different alert levels, ranging from stage 1 (open) to stage 5 (lockdown) (63). According to these triggers, the city enacted stage 5 between June 26, 2020 and July 26, 2020 to mitigate the summer surge, and between December 23, 2020 and February 9, 2021 to mitigate the winter surge, with the COVID-19 ICU census peaking on January 12, 2021 at 190, just short of the estimated local capacity of 200 patients. Austin opened an alternative care site in a large convention center on January 9 and triggered the state’s GA-32 order which restricted restaurant capacity and elective surgeries on January 10, after COVID-19 patients exceeded 15% of all hospitalized patients in the region for seven consecutive days (64–66). The estimated reproduction number declined throughout the stage 5 period, reaching a minimum of 0.65 (95% CrI: 0.5 to 0.9) on February 2.

Population mobility, as measured by the proportion of the day spent at home and numbers of visits to public points of interest, declined sharply during the spring 2020 shelter-in-place order, and then exhibited fluctuations that tracked local COVID-19 policies and epidemiological trends (Fig. 2*B*). After reducing the dimensionality of eight mobility variables via a principal components analysis, we find that the first principal component clearly reflects known holidays and other anomalous periods, including Thanksgiving, Christmas, and the catastrophic Texas winter storm of February 2021 which forced many residents to shelter in place (67). The academic calendars of the local K-12 school districts and the University of Texas at Austin are reflected in the changing frequency of visits to campuses but have little impact on the overall mobility trends reflected in the principal components analysis (*SI Appendix*, Fig. S6). Fluctuations in bar and restaurant visits likewise mirror changing COVID-19 restrictions.

When a community adopts precautionary measures that reduce transmission risks in public venues—-like face masking, keeping physical distance, and proactive testing—-the relationship between mobility and transmission may weaken; the same level of mobility may correspond to a lower level of transmission. When communities loosen such measures, the reverse may occur. We indirectly estimate changes in such precautionary behavior by simulating a counterfactual scenario in which the relationship between mobility and transmission is fixed at the level estimated from the 4 wk beginning on March 13, 2020, the day of the first reported hospital admission. By comparing the resulting hypothetical transmission rates to those originally observed, we estimate the changing relationship between mobility and transmission (Fig. 2*D*). We estimate that, on February 14, 2021, mobility-associated transmission was reduced by 62% (95% CrI: 52 to 68%) relative to early 2020.

We estimate that 15.9% (95% CrI: 15.6 to 16.4%) of the population had been infected by the end of February 2021, and validate these results with CDC seroprevalence estimates (Fig. 2*E*) (48). The estimated prevalence of SARS-CoV-2, including asymptomatic infections, peaked at 0.8% (0.7 to 0.9%) in early January 2021 (*SI Appendix*, Fig. S9). We estimate the time-varying case detection rate by comparing predicted infections to observed case counts. The rate ranged from just under 25% in March 2020 to a peak of 70% in December 2020 (8) (Fig. 2*F*). On February 1, 2021, the city reported almost 6,000 previously unreported cases dating back several months; 2 wk later, reporting was largely suspended as a historic freeze brought the city to a halt (50, 51).

**Fig. 3.**
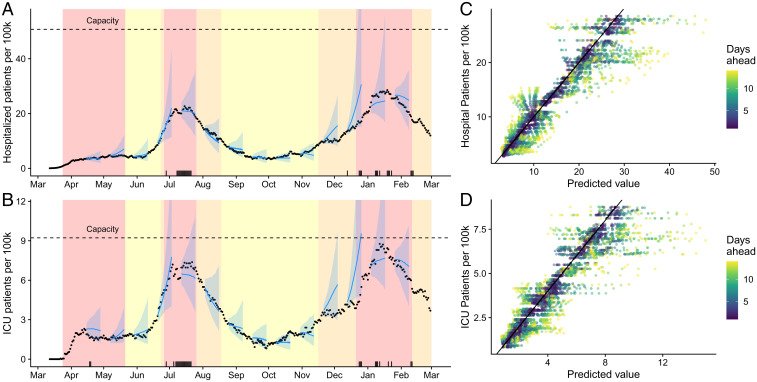
Retrospective validation of Austin area COVID-19 hospitalization and ICU projections from March 12, 2020 through February 1, 2021. (*A* and *B*) A comparison of predicted and observed (*A*) COVID-19 hospital census and (*B*) COVID-19 ICU census. Blue lines and points provide 2-wk-ahead projections with 95% prediction intervals at 14 time points throughout the pandemic; black points are reported data. The black tick marks along the bottom indicate our 30 worst forecasts, that is, dates with large differences between the observed value and our 2-wk-prior prediction of the value. (*C* and *D*) Predicted (median) versus observed (*C*) hospital or (*D*) ICU COVID-19 census. Colors indicate the time horizon of each prediction; the diagonal line indicates that the predicted value equals the observed value.

Since May 29, 2020, we have used this model on a daily basis to provide 3-wk-ahead projections of COVID-19 healthcare demand on a dashboard that is widely used by local policy makers, healthcare systems, press, and the public (47). In retrospective validation, we find that 92.9%, 89.5%, and 87.9% of reported daily COVID-19 hospital census values fall within the 95% prediction intervals of our 1-wk-, 2-wk-, and 3-wk-out projections, respectively (Fig. 3). For COVID-19 ICU data, the corresponding performance metrics are 89.7%, 88.1%, and 87.0%. Our models tend to overproject COVID-19 healthcare demand, particularly at pandemic peaks (Fig. 3, black tick marks). During the summer and winter peaks, the forecasts indicated that the city might exhaust local ICU capacity but not hospital general bed capacity.

**Fig. 4.**
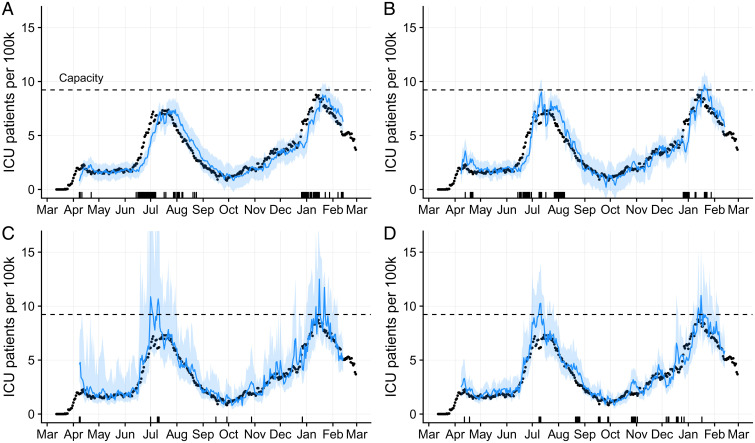
Comparison of 1-wk-ahead COVID-19 ICU projections for four models, from April 1, 2020 through February 1, 2021. Observed data (black points) are superimposed on forecasts using (*A*) a random walk model, (*B*) an autogenerated ARIMA model, (*C*) a simplified version of our model omitting the mobility covariate, and (*D*) the full version of our model. Blue lines and shading represent medians and 95% prediction intervals, respectively, across 1,000 stochastic projections. The tick marks on the *x* axis indicate days on which the observed ICU usage fell outside of the 1-wk-ahead 95% prediction interval. The horizontal dashed line indicates the estimated ICU capacity of 200 beds for the Austin metropolitan area.

We compare the forecasting performance of our model to three alternative models—a simple random walk (68), an automated autoregressive integrated moving average (ARIMA) model (68), and a simple version of our model which omits the mobility covariate (Fig. 4). The proportion of observed data points that fall within the 95% prediction intervals is highest for the nonmobility version of our model, across the 1-wk, 2-wk, and 3-wk forecasting horizons (*SI Appendix*, Fig. S10). Our full model performs on par with the ensemble model from the CDC’s national COVID-19 healthcare forecasting hub (69) and outperforms the simpler random walk and ARIMA models (*SI Appendix*, Fig. S10*A*). The four models achieve comparable levels of error in their (median) point estimates (*SI Appendix*, Fig. S10*B*). However, these summary statistics do not reflect time-dependent performance differences among the models. Our full model offers the highest precision and accuracy during pandemic surges (Fig. 4 and *SI Appendix*, Figs. S11 and S12). Although the two simple statistical models offer highly accurate (and precise) forecasts during periods of relative stability, they fail to predict exponential growth and rapid decline. Our model outperforms the nonmobility version in reducing uncertainty—providing narrower prediction intervals—particularly at critical epidemic change points.

## Discussion

Through a unique collaboration between policy makers, public health officials, healthcare systems, and scientists in the Austin–Round Rock metropolitan area, we developed a flexible model for pandemic surveillance and healthcare forecasting that has guided local COVID-19 responses for over a year. Daily projections have contributed to key pandemic decisions, including enacting the initial Stay Home–Work Safe order (52), face mask mandates (57), and the launch of an alternate care facility to accommodate healthcare overflow (66). Throughout the pandemic, city leadership and local news organizations have regularly cited our model outputs to communicate risks and explain policy changes to the public (70–72).

Although early COVID-19 risk assessments and forecasts relied almost exclusively on COVID-19 case and mortality data, we find that COVID-19 hospital admissions provide a more accurate and timely indication of recent transmission and imminent healthcare usage. Given the average 5.2 d between infection and symptom onset and average 5.9 d from symptom onset to hospital admission, we expect hospital admissions data to lag infection by roughly 11 d to 12 d, although there is significant individual variation in the time course of infection (73, 74). Case counts could provide a more immediate signal of incidence, if cases seek testing and receive rapid results immediately after or even before symptom onset. However, testing in the United States has been plagued by biases and delays throughout the pandemic, including restricted access (75, 76), public health guidance to wait until after symptom onset (77), and chronic lags in laboratory processing and reporting (77, 78). We expect case data to exhibit 11- to 12-d lags similar to hospital admissions data, given the sequence of delays from infection to symptom onset to test seeking to receipt of test results. A national survey in September 2020 suggested that cases seek tests an average of 2.5 d after first symptoms and wait an average of 3.7 d to receive results (78). Moreover, case count data have persistently exhibited racial, ethnic, and geographic biases due to differential testing access and availability (27). Thus, hospital admissions provide an equally lagged but potentially less biased signal of recent transmission than case data. Despite the utility of COVID-19 hospital admission counts, such data were not widely available in the United States until 9 mo into the pandemic (26). Part of the challenge is that COVID-19 status is not always known at the time of admission, particularly early in the pandemic, when diagnostic resources were limited (75). In Austin, hospitals occasionally updated admissions counts retroactively when SARS-CoV-2 confirmations were delayed.

We estimate that, early in the pandemic, the SARS-CoV-2 reproduction number (*R_t_*) reached 5.8 (95% CrI: 3.6 to 7.9). Although high, it is consistent with previously published estimates (79). Similar estimates in other cities have been attributed to superspreading events, which we do not explicitly model (80). We note that our estimate is sensitive to the timing of COVID-19 emergence in Austin. If we assume that the initial case arrived on January 20, 2020 rather than February 19, 2020 (which is based on the timing of the first COVID-19 hospital admission), then we estimate a maximum *R_t_* of 4.5 (95% CrI: 3.0 to 6.4). However, the estimates quickly converge after March 13, 2020, when COVID-19 healthcare data become available (*SI Appendix*, Fig. S5).

We estimate that the case detection rate has been highly variable, ranging from less than 20% of cases reported at the outset to well over half reported since early 2021. This variation likely reflects evolving testing priorities, technologies, and access, as well as changes in test seeking behavior driven by fear and effective public health communications (81). However, these citywide averages do not capture demographic and geographic heterogeneity in testing behavior (27, 81). For example, children are much less likely to develop symptoms and seek testing than adults, although some private schools have mandated weekly or more frequent testing of all students and staff. The University of Texas at Austin population is similarly overrepresented in the citywide testing data, with their proactive testing program screening an average of 340 students and faculty per day during the 2020–2021 academic year (82).

Our retrospective estimates of COVID-19 infections in Austin are consistent with seroprevalence data (48). Just prior to the summer 2021 emergence of the Delta variant in Austin, we estimated that just under 20% of the Austin-area population had been infected and 58% of adults over age 16 y had received at least one dose of a SARS-CoV-2 vaccine (83, 84). As vaccine uptake counterbalances increased transmissibility of COVID-19 variants, our model can be used to continually monitor local transmission dynamics. Going forward, forecasting models like ours must integrate the dynamics of infection-acquired and vaccine-acquired immunity against wild-type and variant SARS-CoV-2 viruses.

Our forecasting model performs well in comparison to simpler mechanistic and nonmechanistic statistical models. Although the four models considered achieve comparable coarse-grained performance statistics, our mobility-driven mechanistic model provides the best combination of accuracy and precision surrounding pandemic surges, when reliable forecasts are particularly important for effective healthcare provisioning, public health responses, and general risk awareness. Removing the mobility covariate from our model significantly increases forecasting uncertainty. Although this increases coverage—the proportion of observed values falling within prediction intervals—it significantly reduces the informativeness and public health utility of the forecasts. Since May 2020, our model projections have informed numerous time-sensitive policy decisions and response actions, including resource planning by local hospitals, urgent requests to state and federal agencies for additional surge resources, the launch and dismantling of alternative care sites to provide additional healthcare capacity, and numerous changes in the Austin-area COVID-19 alert stage to communicate and manage rising and declining risks (43).

In March 2020, we faced an unexpected technical challenge. Prior to the COVID-19 pandemic, most models of respiratory virus transmission assumed that daily contact patterns would be fairly stable. The simplest models assumed that populations are entirely homogeneous and well mixed, others incorporated age-specific contact patterns from diary-based surveys (85) or inferred from epidemiological data (86), and still others assumed complex networks of interactions based on sociological data sources (87, 88). The nationwide shelter-in-place orders broke these assumptions. The cell phone mobility data provided by SafeGraph and other technology companies provided an immediate and valuable window into changing behavioral patterns (19). Early in the pandemic, cell phone GPS data reflected COVID-19 policies and correlated with transmission rates (18, 89). Our model comparison—with and without mobility data—further suggests that mobility data can provide an immediate and reliable indication of changing risk behavior. However, the relationship between mobility and transmission can evolve as communities adopt and relax precautionary behavior. To capture this, we estimated a coefficient that relates daily mobility to daily transmission rates in Austin. The data suggest that mobility-associated risks of transmission initially declined in the spring of 2020, then spiked following the White House’s Opening Up America Again campaign, and slowly increased between August and the end of 2020. As novel sources of behavioral information become available, such as more granular mobility trends (90), Bluetooth-enabled contact tracing records (91), or self-reported face covering usage (22), we should carefully consider and (if possible) explicitly model the observational processes used to collect the data and the behavior dynamics that shape them.

Our retrospective analysis of the Austin experience provides anecdotes regarding the impact of COVID-19 policies on risks. Notably, the statewide reopening in May 2020 appeared to fuel the major summer wave. The constellation of policy relaxation, behavioral fatigue, return to school, and winter holidays preceded the winter surge. Recent studies have quantified the impact of restaurant and bar restrictions, school closures, and mask mandates on local SARS-CoV-2 transmission (92–94). Our study of the COVID-19 pandemic in Austin does not disentangle the relative impacts of such measures but provides an intuitive case study for the dynamic interplay between public policy, human behavior, and viral transmission.

Throughout the pandemic, we have applied this model to provide estimates of key COVID-19 indicators and month-ahead hospitalization forecasts. In April 2020, we started by providing model-based projections at the city task force meetings multiple times per week. By June 2020, we had automated the data processing and statistical fitting procedures and launched a public-facing dashboard (47). The choice of indicators and plotting formats were honed through months of engagements with city leadership and local media. The Austin–Round Rock MSA dashboard provides the daily reproduction number with 95% CrIs, the probability that the pandemic is in a growth phase (that is, the probability that the reproduction number is above one), and the 14-d change in incidence as a percent (*SI Appendix*, Figs. S14 and S15). It also includes time-series graphs for COVID-19 hospital admissions, hospital census, and ICU census, each of which displays data from the beginning of the pandemic and spaghetti plot forecasts, which convey uncertainty by depicting 100 distinct stochastic projections. This visually communicates that qualitatively different futures may be equally likely, and emphasizes the considerable uncertainty we have faced throughout the pandemic stemming from data quality issues and our inability to anticipate changes in behavior and government policies. Our retrospective performance evaluation revealed that the 95% prediction intervals do not capture 95% of the future data. Specifically, the model failed to predict the rapid deceleration of transmission leading to the peaks observed in July and January. One possible explanation is unmodeled feedback from the system (Austin) to the model, as suggested in prior COVID-19 forecasting studies (95). As COVID-19 hospitalizations climbed, city leadership enacted stricter policies and aggressively communicated the pessimistic forecasts to the public to encourage precautionary behavior and curb transmission. Indeed, our largest prediction errors are clustered around the two pandemic peaks, shortly after Austin transitioned to its most restrictive COVID-19 alert stage. The model does not directly or immediately capture such policy and behavioral changes but rather estimates their effects, with delay, from mobility and hospitalization data. Our COVID-19 forecasting successes and failures will likely inspire a new generation of epidemiological models that include mechanistic behavioral dynamics, organizational decision-making, and feedback between sociological and epidemiological dynamics.

Through discussions with the city’s COVID-19 task force, media outlets in central Texas, local school districts and universities, major hospital systems, and community organizations, we believe that the dashboard has served as a trusted, daily touchstone for the leadership and residents of Austin, TX. For example, the modeling informed decisions to enact the city’s Stay Home–Work Safe order in March 2020, the design of the staged alert system that has guided policy since May 2020 (43), the provisioning of hotel rooms as isolation facilities for populations experiencing homelessness and university students living in congregant settings (96), and the launch of an alternative care site at the convention center to accommodate healthcare overflow, as well as reopening policies by universities and schools throughout the city (97). Arguably, the primary value of this effort has been providing a common, predictive understanding of the changing risks, even when the forecasts have been imperfect.

We note three key limitations of our model. First, we do not consider superspreading events, which could lead our model to underestimate future risks, particularly if a superspreading event occurs in a long-term care facility (98). Our model likely captures the potential for sudden transmission rate changes from superspreading events; however, mechanistically incorporating such dynamics could increase the precision of our projections. Second, we assume that Austin is a well-mixed population, and thus ignore important heterogeneities such as long-term care facilities (99) and the extreme east–west segregation of the city, with majority-Latino communities experiencing much higher rates of infection and severe outcomes than the majority-White communities (100–104). Incorporating such heterogeneity for Austin and carefully adapting such assumptions to other cities could substantially improve projections and inform more strategically targeted mitigation efforts. Finally, our estimates for SARS-CoV-2 incidence are sensitive to the assumed infection hospitalization rates, which vary across age and health subgroups and remain uncertain (37, 105). Incorporating uncertainty in these parameters would yield wider and, arguably, more reasonable credibility intervals around our estimates for SARS-CoV-2 incidence and case reporting rates. As better data become available, through serological surveys and prospective studies, these parameters can be readily updated.

Immediate, reliable, and comprehensive access to SARS-CoV-2 hospitalization, vaccination, and molecular surveillance data—-all of which are collected in electronic databases throughout the United States—-is critical for real-time risk assessments, reliable forecasting, and, most important, effective decision-making by individuals, organizations, and government agencies. Translating such data into interpretable indicators and accessible graphs can improve coordination among stakeholders and encourage public buy-in. Our model is designed to provide such retrospective insight and actionable guidance for the public and policy makers in communities throughout the United States.

## Materials and Methods

### Epidemiological Model.

We use an age- and risk-structured SEIR model that incorporates asymptomatic and symptomatic transmission, hospitalization, and mortality. The demographic and risk structure are based on estimates for the Austin–Round Rock MSA (*SI Appendix*, Fig. S2 and Tables S4–S6), and the natural history of SARS-CoV-2 follows published estimates (*SI Appendix*, Tables S1–S3). Transmission rates are driven by regional mobility, and the governing relationship between mobility and transmission is allowed to change daily to reflect the dynamic impacts of policy and behavior. The hospital stay duration is also allowed to vary as standards of care and healthcare strain impact the COVID-19 hospital experience (106, 107).

The model structure is diagrammed in *SI Appendix*, Fig. S1, and we present the stochastic formulation in the equations below. For each age and risk group, we build a separate set of compartments to model the transitions between the states: susceptible (*S*), exposed (*E*), presymptomatic infectious (*P^Y^*^)^, preasymptomatic infectious (*P ^A^*), symptomatic infectious (*I^Y^*^)^, asymptomatic infectious (*I^A^*), symptomatic infectious that are hospitalized (*I^H^*), recovered (*R*), and deceased (*D*). The symbols *S*, *E*, *P^Y^*^,^ *P^A^*, *I^Y^*^,^ *I^A^*, *I^H^*, *R*, and *D* denote the number of people in that state in the given age/risk group, and the total size of the age/risk group is

$$N=S+E+P^{Y}+P^{A}+I^{Y}+I^{A}+I^{H}+R+D.$$

Transitions between compartments are governed using the tau-leap method (108, 109) with key parameters given in *SI Appendix*, Tables S1–S3. The stochastic model for individuals in age group and risk group is given by

$$\begin{array}{l} S_{a,r}(t+1)-S_{a,r}(t)=-P_{1}\\ E_{a,r}(t+1)-E_{a,r}(t)=P_{1}-P_{2}\\ P_{a,r}^{A}(t+1)-P_{a,r}^{A}(t)=(1-\tau )P_{2}-P_{3}\\ P_{a,r}^{Y}(t+1)-P_{a,r}^{Y}(t)=\tau P_{2}-P_{4}\\ I_{a,r}^{A}(t+1)-I_{a,r}^{A}(t)=P_{3}-P_{5}\\ I_{a,r}^{Y}(t+1)-I_{a,r}^{Y}(t)=P_{4}-P_{6}-P_{7}\\ I_{a,r}^{H}(t+1)-I_{a,r}^{H}(t)=P_{7}-P_{8}-P_{9}\\ R_{a,r}(t+1)-R_{a,r}(t)=P_{5}+P_{6}+P_{8} \end{array}$$

with

$$\begin{array}{l} P_{1}∼B(n=S_{a,r}(t)\text{,}p=1-{\left(1-F_{a,r}(t)\right)}^{dt})\\ P_{2}∼B(n=E_{a,r}(t)\text{,}p=1-{\left(1-\sigma \right)}^{dt})\\ P_{3}∼B(n=P_{a,r}^{A}(t)\text{,}p=1-{\left(1-\rho^{A}\right)}^{dt})\\ P_{4}∼B(n=P_{a,r}^{Y}(t)\text{,}p=1-{\left(1-\rho^{Y}\right)}^{dt})\\ P_{5}∼B(n=I_{a,r}^{A}(t)\text{,}p=1-{\left(1-\gamma^{A}\right)}^{dt})\\ P_{6}∼B(n=I_{a,r}^{Y}(t)\text{,}p=1-{\left(1-(1-\pi )\gamma^{Y}\right)}^{dt})\\ P_{7}∼B(n=I_{a,r}^{Y}(t)\text{,}p=1-{\left(1-\pi \eta \right)}^{dt})\\ P_{8}∼B(n=I_{a,r}^{H}(t)\text{,}p=1-{\left(1-(1-\nu )\gamma^{H}(t)\right)}^{dt})\\ P_{9}∼B(n=I_{a,r}^{H}(t)\text{,}p=1-{\left(1-\nu \mu (t)\right)}^{dt}), \end{array}$$

where *B*(*n*, *p*) denotes a binomial distribution with *n* trials each with probability of success *p*; *γ^A^*, *γ^Y,^* and $\gamma^{H}(t)$ are the recovery rates for the *I^A^*, *I^Y^*^,^ and *I^H^* compartments, respectively; *σ* is the exposed rate; *ρ^A^* and *ρ^Y ^*are the pre(a)symptomatic rates; *τ* is the symptomatic ratio; *π* is the proportion of symptomatic individuals requiring hospitalization; *η* is the rate at which hospitalized cases enter the hospital following symptom onset; *ν* is mortality rate for hospitalized cases; and μ(t) is the daily instantaneous rate at which terminal patients die.

$F_{a,r}$ denotes the force of infection for individuals in age group *a* and risk group *r* and is given by

$$\begin{array}{l} F_{a,r}(t)=\;\sum_{i\in A}\sum_{j\in K}(I_{i,j}^{Y}(t)\omega^{Y}+I_{i,j}^{A}(t)\omega^{A}+\\ P_{i,j}^{Y}(t)\omega^{PY}+P_{i,j}^{A}(t)\omega^{PA})\cdot (\beta (t)\phi_{a,i}/N_{i}), \end{array}$$

where *A* and *K* describe the age and risk groups, respectively; *ω^A^*, *ω^Y,^ ω^PA^*, and *ω^PY ^*are the relative infectiousness of the *I^A^*, *I^Y^*^,^ *I ^PA^*, and *I^PY^* compartments, respectively; and $\phi^{a,i}$ is the mixing rate between age group *a* and age groups i∈A.

We define the time-dependent transmission rate β(t) as a function of mobility as

$$\begin{array}{l} \beta (t)=\beta (0)\cdot e^{b_{1}(t)\cdot PC1(t)+b_{2}(t)\cdot PC2(t)+Z(t)}\\ b_{1}(t)∼N(b_{1}(t-1),\sigma_{b_{1}}\sqrt{dt})\\ b_{2}(t)∼N(b_{2}(t-1),\sigma_{b_{2}}\sqrt{dt})\\ Z(t)∼N(\psi \cdot Z(t-1),\sigma_{Z}\sqrt{dt}), \end{array}$$

where $b_{1}(0)=0,\;b_{2}(0)=0,\;Z(0)=0$, *PC*1 and *PC*2 describe the first and second principal components from our mobility data as described below, ψ=0.97, and $N(\mu_{N},\sigma_{N})$ denotes a normal distribution with mean of *μ_N_* and SD of *σ_N_*.

Finally, we allow the duration in the hospital for individuals who survive, $\gamma^{H}(t)$, and those who pass away, μ(t), to vary in time as

$$\begin{array}{l} \mu (t)=\mu (0)\cdot e^{Z_{\mu }}\\ Z_{\mu }(t)∼N(\psi_{\mu }\cdot Z_{\mu }(t-1)\sigma_{\mu }\sqrt{dt})\\ \gamma^{H}(t)=\gamma^{H}(0)\cdot e^{Z_{\gamma }}\\ Z_{\gamma }(t)∼N(\psi_{\gamma }\cdot Z_{\gamma }(t-1),\sigma_{\gamma }\sqrt{dt}), \end{array}$$

where $Z_{\mu }(0)=0,\;Z\gamma (0)=0$, and $\psi_{\gamma }=0.99$. To run the SEIR model without mobility, we set PC1(t)=0 and PC2(t)=0 for all *t*, so $\beta (t)=\beta (0)\cdot e^{Z(t)}$.

### Mobility Trends.

We used mobility trends data from the Austin MSA to inform the transmission rate in our model. Specifically, we ran a principal component analysis (PCA) on eight independent mobility variables provided by SafeGraph (19), including 1) home dwell time and visits to 2) universities, 3) bars, 4) grocery stores, 5) museums and parks, 6) medical facilities, 7) schools, and 8) restaurants. All metrics are provided at the census block group (CBG) and aggregated to the five county metropolitan regions (Bastrop, Caldwell, Hays, Travis, and Williamson Counties). For each CBG, SafeGraph provides the daily average home dwell time and number of reporting devices. We estimate average home dwell time in the MSA by averaging across CBGs weighted by the number of reporting devices. For all other visitation metrics, we sum the total visits for the specific indicator across all CBGs within the MSA. We baseline each metric according to prepandemic mobility by calculating the average value for the metric in the MSA from January and February of 2020 and dividing all subsequent values of that metric by the prepandemic baseline. We carry out a PCA on the eight baselined metrics using all data up to the day the projections are made, which captures almost as much variation in mobility as a more granular sliding window PCA (*SI Appendix*, Fig. S7). We use the first two principal components as covariates for a regression as described in the modeling equations for β(t). Daily 7-d averages for the raw mobility data can be seen in *SI Appendix*, Fig. S6.

### Model Fitting.

We obtained daily hospital admit, discharge, census, and death data for the Austin MSA from Austin Public Health. We assumed all sources of data were negative binomially distributed around their predicted values from the SEIR stochastic model with dispersion parameter *k*. We chose informative but relatively dispersed priors for certain parameters for stability in parameter estimation and to prevent the model from overfitting data through large perturbations to time-dependent variables. A full explanation of the likelihood for the model can be found in *SI Appendix*. We estimated $\beta (t),\;\gamma^{H}(t),\;\mu (t)$ *k*, *σ_Z_*, $b_{1}(t),\;b_{2}(t),\;\sigma_{b_{1}},\;\sigma_{b_{2}},\;\psi_{\mu },\;\sigma_{\mu }$, and $\sigma_{\gamma }$ and fixed the remaining parameters as described in *SI Appendix*, Tables S1–S3.

Fitting was carried out using the iterated filtering algorithm made available through the mif2 function in the pomp package in R (110–112). This algorithm is a stochastic optimization procedure; it performs maximum likelihood estimation using a particle filter to provide a noisy estimate of the likelihood for a given combination of the parameters. For each parameter combination, we ran 300 iterations of iterated filtering with a cooling fraction of 50% every 60 steps, each with 3,500 particles. This iterated filtering was run 50 times, and the maximum likelihood estimate (MLE) among these 50 was selected. We calculated smoothed posterior estimates for all of the states within the model through time (including β(t) and other time-dependent parameters which are technically state variables in our model formulation). We estimated these smoothed posteriors as follows:

1) We ran 1,000 independent particle filters at the MLE, each with 2,500 particles. For each run, *l*, of particle filtering, we kept track of the complete trajectory of each particle, as well as the filtered estimate of the likelihood, *L_l_*.
2) For each of the 1,000 particle filtering runs, we randomly sampled a single complete particle trajectory, giving us 1,000 separate trajectories for all state variables.
3) We resampled 1,000 trajectories from these 1,000 trajectories with probabilities proportional to *L_l_* to give a distribution of state trajectories.

The result can be thought of as an empirical Bayes posterior distribution; that is, a set of 1,000 smoothed posterior draws from all state variables, conditional on the MLEs for the model’s free parameters. This smoothed posterior distribution is how we calculate summary statistics for our time-varying state variables. Our estimates for β(t) are converted to *R*(*t*) estimates as described below, and model estimates for the instantaneous discharge rates for surviving ($\gamma^{H}(t)$) and dying (μ(t)) patients can be found in *SI Appendix*, Fig. S13.

### Making Projections.

Our model fitting procedure provides MLE for all of the key parameters (e.g., the SD governing the random walk of the transmission rate) in the model alongside smoothed posteriors for the state variables (e.g., the number of individuals in each compartment of the model or the daily transmission rate). We sample from the smoothed posterior distribution to obtain a distribution of initial state conditions for the projections. We initialize 1,000 projections with those initial state conditions and run the stochastic model forward according to the MLE of the fixed parameters. In this way, we capture two sources of uncertainty in our parameter estimates: 1) uncertainty in the underlying state of the community at the time the projections are made and 2) uncertainty in how behavior might change in the future as captured by the random walk function in our transmission rate.

### Projection Model Comparison.

We compare projections from the SEIR epidemiological model with projections from statistical null models provided by the forecast package in R (68). For the random walk model, we use an ARIMA model of order (p=0,d=1,q=0) (68), and we use the Hyndman–Khandakar algorithm for automatically determining the order of an ARIMA model for the Auto ARIMA model (68). We fit the models to all available data up to the date the projection is made, and project forward with the fitted model.

### Interpreting Model Outputs.

#### Time-varying reproduction number (*R_t_*).

To estimate the time-varying reproduction number (*R_t_*), we apply the next-generation method to our daily estimated smoothed posterior distributions for β(t) with the MLE values of the estimated parameters and the fixed parameters listed in *SI Appendix*, Tables S1–S3 (113).

#### Reporting rates.

We estimate the reporting rates by comparing our estimates for daily incidence with daily reported cases counts for the Austin MSA (Bastrop, Caldwell, Hays, Travis, and Williamson Counties) as provided by *The New York Times* (8). To roughly estimate changing reporting rates, we lag the case data by 11 d to account for the lag between infection and case reporting (73, 78). In estimating the maximum and minimum reporting rates, we exclude case data for February 2021, because reporting was impacted by a severe weeklong winter freeze and the reporting of a large number of backlogged cases (49–51).

#### Estimating Austin COVID-19 seroprevalence.

COVID-19 seroprevalence estimates are not available for the Austin metropolitan region, but the CDC has conducted biweekly Texas seroprevalence estimates since the summer of 2020 (48). We adjust the Texas seroprevalence estimates to account for the heterogeneous burden of the pandemic across the state. Specifically, we assume that Austin seroprevalence can be estimated as

$$I_{\text{austin}}=I_{\text{texas}}\cdot \frac{D_{\text{austin}}}{D_{\text{texas}}},$$

where $I_{\text{texas}}$ is the seroprevalence estimate provided by the CDC for the state of Texas, and *D* indicates the per capita mortality rate for Austin or the state of Texas as provided by *The New York Times* (8). As carried out in ref. 48, we shift all time-dependent estimates to their corresponding date of infection, so seroprevalence estimates are shifted to 7 d before the first sampling day to account for the time it takes to become seropositive following infection, and mortality date are shifted 20 d to account for the delay between infection and mortality (114). We then compare the corrected estimate for $I_{\text{austin}}(t)$ with the daily cumulative estimated infections from the model.

#### Estimating the time-varying relationship between mobility and transmission.

Our model estimates the time-varying transmission rate as

$$\beta (t)=\beta (0)\cdot e^{b_{1}(t)\cdot PC1(t)+b_{2}(t)\cdot PC2(t)+Z(t)},$$

with $b_{1}(t)$ and $b_{2}(t)$ governing the relationship between the mobility data and the transmission rate. Since transmission is governed by a combination of $b_{1}(t),\;b_{2}(t)$, and *Z*(*t*), an increase in one may be compensated by a decrease in another without significantly changing the overall transmission rate. Thus, we cannot easily estimate the contribution of each in isolation. Instead, we estimate the time-varying relationship between mobility and transmission through a comparison between our fitted model and a counterfactual scenario where $b_{1}(t),\;b_{2}(t)$, and *Z*(*t*) are fixed at their average initial estimated values ($\bar{b_{1}},\;\bar{b_{2}}$, and $\bar{Z}$). Specifically, we estimate $\bar{b_{1}},\;\bar{b_{2}}$, and $\bar{Z}$ as the average for the respective parameters over the first 4 wk of hospitalization data from the fitted model (from March 13, 2020 to April 10, 2020), and calculate the expected transmission rate based on this initial relationship and subsequent mobility data as

$$\beta \prime (t)=\beta (0)\cdot e^{\bar{b_{1}}\cdot PC1(t)+\bar{b_{2}}\cdot PC2(t)+\bar{Z}}.$$

β′(t) can be thought of as the counterfactual transmission rate if the initial relationship between mobility and transmission remained constant over the course of pandemic. We estimate the reduction in mobility-driven transmission that is unexplained by mobility levels as

$$\frac{\beta (t)-\beta \prime (t)}{\beta \prime (t)}.$$

We provide a point estimate for the overall reduction in mobility–transmission risk on February 14, 2021 relative to early in the pandemic, and provide a sensitivity analysis with respect to the start and duration of the baseline period (*SI Appendix*, Fig. S8).

## Supplementary Material

Supplementary File

## Data Availability

All code and healthcare time-series data used in this study are publicly available and have been deposited in GitHub (https://github.com/UT-Covid/SEIR-Austin).
