# Supplementary material for: Real-time pandemic surveillance using hospital admissions and mobility data
Source: Proc Natl Acad Sci U S A. 2022 Feb 1;119(7):e2111870119. doi: 10.1073/pnas.2111870119 (PMC8851544; doi:10.1073/pnas.2111870119)
Supplement: Supplementary File [file pnas.2111870119.sapp.pdf]

Supplementary Information: Real-time pandemic surveillance using hospital admissions and mobility data

Spencer J. Fox*, Michael Lachmann*, Mauricio Tec, Remy Pasco, Spencer Woody, Zhanwei Du, Xutong Wang, Tanvi A. Ingle, Emily Javan, Maytal Dahan, Kelly Gaither, Mark E. Escott, Stephen I. Adler, S. Claiborne Johnston, James G. Scott, Lauren Ancel Meyers

* contributed equally

Epidemiological model diagram

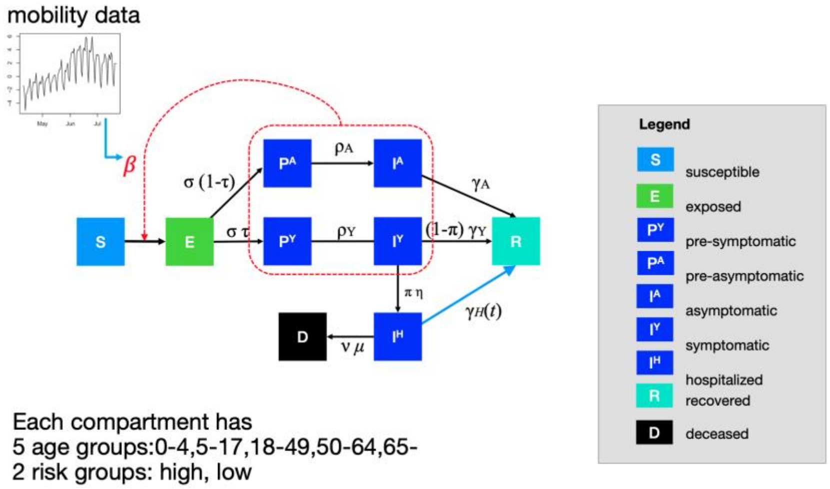

Figure S1. Compartmental model of COVID-19 transmission in the Austin MSA. Each subgroup (defined by age and risk) is modeled with a separate set of compartments. Upon infection, susceptible individuals (*S*) progress to exposed (*E*) and then to either pre-symptomatic infectious (P^Y) or pre-asymptomatic infectious (P^A) from which they move to symptomatic infectious (I^Y) and asymptomatic infectious (I^A) respectively. All asymptomatic cases eventually progress to a recovered class where they remain protected from future infection (*R*); symptomatic cases are either hospitalized (I^H) or recover. Mortality (*D*) varies by age group and risk group and is assumed to be preceded by hospitalization.

Table S1. Model parameters[a]

Parameters	Value	Source
Start date	February 19, 2020	Estimated
Initial infections	1 symptomatic case age 18-49y	Assumption
$\beta(t)$: daily transmission rate	N/A	Estimated
γ^A : recovery rate on asymptomatic compartment	Equal to γ^Y	Assumption
γ^Y: recovery rate on symptomatic non-treated compartment	0.25	He et al. (1)
τ: symptomatic proportion (%)	57	Gudbjartsson et al. (2)
σ: exposed rate	1/2.9	Zhang et al. (3); He et al. (1)
ρ^A: pre-asymptomatic rate	Equal to ρ^Y	Assumption
ρ^Y: pre-symptomatic rate	$\frac{1}{2.3}$	He et al. (1)
P: proportion of pre-symptomatic transmission	44%	He et al. (1)
ω^P: relative infectiousness of pre-symptomatic individuals	$\omega^P = \frac{P}{1-P} \frac{\tau\omega^Y[YHR/\eta + (1-YHR)/\gamma^Y] + (1-\tau)\omega^A/\gamma^A}{\tau\omega^Y/\rho^Y + (1-\tau)\omega^A/\rho^A}$ $\omega^{PY} = \omega^P\omega^Y, \omega^{PA} = \omega^P\omega^A$	Assumption
ω^A : relative infectiousness of infectious individuals in compartment I^A	$\frac{2}{3}$	He et al. (4)
IFR: infected fatality ratio, age specific (%)	Low risk: [0.0009, 0.002, 0.034, 0.25, 0.64]<br>High risk: [0.009, 0.02, 0.34, 2.52, 6.44]	Age adjusted from Verity et al. (5)
YFR: symptomatic fatality ratio, age specific (%)	Low risk: [0.0016, 0.0038, 0.060, 0.44, 1.13]<br>High risk: [0.016, 0.038, 0.59, 4.4, 11.3]	$YFR = \frac{IFR}{\tau}$
h: high-risk proportion, age specific (%)	[8.3, 14.1, 16.5, 33, 47]	Estimated using 2015-2016 Behavioral Risk Factor Surveillance System (BRFSS) data as described below (6–8)

[a]Values given as five-element vectors are age-stratified with values corresponding to 0-4, 5-17, 18-49, 50-64, 65+ year age groups, respectively.

Table S2. Hospitalization parameters

Parameters	Value	Source
$\gamma^H(t)$: recovery rate in hospitalized compartment	Fitted	
YHR: symptomatic case hospitalization rate (%)	Low risk: [0.04, 0.03, 1.9, 4.1, 4.9]<br>High risk: [0.4, 0.3, 19.0, 41.1, 48.8]	Age adjusted from Verity et al. (5)
π: rate of symptomatic individuals go to hospital, age-specific	$\pi = \frac{\gamma^Y * YHR}{\eta + (\gamma^Y - \eta)YHR}$	
η: rate from symptom onset to hospitalized	0.17	5.9 day average from symptom onset to hospital admission (9)
$\mu(t)$: rate from hospitalized to death	Fitted	
HFR: hospitalized fatality ratio, age specific (%)	[4, 12.4, 3.1, 10.7, 23.2]	$HFR = \frac{IFR}{\tau}$
ν: death rate on hospitalized individuals, age specific	$\nu = \frac{\gamma^H HFR}{\mu + (\gamma^H - \mu)HFR}$	
ICU: proportion hospitalized people in ICU	0.35	Estimated from Austin COVID-19 hospitalization data

Table S3 Contact matrix. Daily number contacts by age group on an average day.

	0-4y	5-17y	18-49y	50-64y	65y+
**0-4y**	1.88	2.02	4.01	0.79	0.28
**5-17y**	0.55	7.06	5.02	0.70	0.22
**18-49y**	0.37	2.19	8.72	1.45	0.21
**50-64y**	0.33	1.62	5.79	2.79	0.50
**65y+**	0.19	0.88	2.36	1.19	1.22

Epidemic starting conditions

We could not estimate the date of the first case directly, since our flexible transmission model can accommodate most dates in early 2020. We therefore used the following independent estimation procedure to select a plausible epidemic start date for Austin. Assuming the parameters given in Tables S1-S3 and a β corresponding to an initial reproduction number of $R_t(0) = 4$ (10), we ran 1,000 stochastic simulations beginning with a single infection. We calculated the number of days between the first infection and the first COVID-19 hospital admission (for all simulations that resulted in hospitalizations). By subtracting the resulting distribution from the date of the first documented COVID-19 hospital admission in Austin (March 13, 2020), we estimate a median epidemic start date of February 19, 2020 (IQR = February 13 - February 25), which is consistent with estimates for the timing of COVID-19 emergence in other US cities (11). We therefore begin all of our projections with a single infected individual arriving on February 19, 2020. Our estimates of R_t throughout the pandemic are robust to this assumption (Figure S5).

Model likelihood

We obtained daily hospital admit ($H_A(t)$), discharge data ($H_L(t)$), total hospitalizations ($H(t)$), and death data ($H_D(t)$) for the Austin MSA from Austin Public Health, and fixed all parameters as indicated in Table S1-S3. We assumed all sources of data were negative binomially distributed around their predicted values from the SEIR stochastic model with dispersion parameter, k, and chose informative, but relatively dispersed priors for certain parameters for stability in parameter estimation and to prevent the model from overfitting data through large perturbations to time-dependent variables. In the end we estimated the following parameters: $\beta(t)$, k, $\sigma_{\mathcal{Z}}$, $b_1(t)$, $b_2(t)$, σ_{b_1}, σ_{b_2}, ψ_μ, σ_μ, σ_γ.

The likelihood for our stochastic model was:

$$p(Y(t), b_1(0), \sigma_{b_1}, b_2(0), \sigma_{b_2}, k|\theta) = p(Y(t)|\theta, b_1(0), \sigma_{b_1}, b_2(0), \sigma_{b_2}, k) \cdot p(\theta, b_1(0), \sigma_{b_1}, b_2(0), \sigma_{b_2}, k)$$

where $Y(t)$ refers to the four types of data from hospitals, θ contains all parameters from Table S1-S3 not explicitly listed, and where

$$p(Y(t)|\theta, b_1(0), \sigma_{b_1}, b_2(0), \sigma_{b_2}, k) = p(H_A(t)|\hat{H}_A(t))p(H_L(t)|\hat{H}_L(t))p(H_D(t)|\hat{H}_D(t))p(H(t)|\hat{H}(t))$$
$$p(\theta, b_1(0), \sigma_{b_1}, b_2(0), \sigma_{b_2}, k) = p(b_1(0)) \cdot p(\sigma_{b_1}) \cdot p(b_2(0)) \cdot p(\sigma_{b_2}) \cdot p(k)$$

with

$$p(H_A(t)|\hat{H}_A(t)) = \binom{k + H_A(t) - 1}{H_A(t)} \cdot p^k(1-p)^{H_A(t)}, \text{ and } p = \frac{k}{k + \hat{H}_A(t)}$$
$$p(H_L(t)|\hat{H}_L(t)) = \binom{k + H_L(t) - 1}{H_L(t)} \cdot p^k(1-p)^{H_L(t)}, \text{ and } p = \frac{k}{k + \hat{H}_L(t)}$$

$$p(H_D(t)|\hat{H}_D(t)) = \binom{k + H_D(t) - 1}{H_D(t)} \cdot p^k(1-p)^{H_D(t)}, \text{ and } p = \frac{k}{k + \hat{H}_D(t)}$$
$$p(H(t)|\hat{H}(t)) = \binom{k + H(t) - 1}{H(t)} \cdot p^k(1-p)^{H(t)}, \text{ and } p = \frac{k}{k + \hat{H}(t)}$$
$$p(b_1(0)) \cdot t_d = \frac{1}{\sqrt{2}} e^{-\frac{1}{2}(\hat{b}_1(0))^2}$$
$$p(b_2(0)) \cdot t_d = \frac{1}{\sqrt{2}} e^{-\frac{1}{2}(\hat{b}_2(0))^2}$$
$$p(\sigma_{b_1}) \cdot t_d = \frac{1}{\Gamma(1.1) \cdot \frac{1}{1.1}^{1.1}} \hat{\sigma}_{b_1}^{1.1-1} e^{-1.1 \cdot \hat{\sigma}_{b_1}}$$
$$p(\sigma_{b_2}) \cdot t_d = \frac{1}{\Gamma(1.1) \cdot \frac{1}{1.1}^{1.1}} \hat{\sigma}_{b_2}^{1.1-1} e^{-1.1 \cdot \hat{\sigma}_{b_2}}$$
$$p(k) \cdot t_d = e^{\hat{k}}$$
and t_d is the number of days in the fitting time period.

Estimation of age-stratified proportion of population at high-risk for COVID-19 complications

We estimate age-specific proportions of the population at high risk of complications from COVID-19 based on data for Austin, TX and Round-Rock, TX from the CDC’s 500 cities project (Figure S2) (12). We assume that high risk conditions for COVID-19 are the same as those specified for influenza by the CDC (6). The CDC’s 500 cities project provides city-specific estimates of prevalence for several of these conditions among adults (13). The estimates were obtained from the 2015-2016 Behavioral Risk Factor Surveillance System (BRFSS) data using a small-area estimation methodology called multi-level regression and poststratification (7, 8). It links geocoded health surveys to high spatial resolution population demographic and socioeconomic data (8).

Estimating high-risk proportions for adults

To estimate the proportion of adults at high risk for complications, we use the CDC’s 500 cities data, as well as data on the prevalence of HIV/AIDS, obesity and pregnancy among adults (Table S4).

The CDC 500 cities dataset includes the prevalence of each condition on its own, rather than the prevalence of multiple conditions (e.g., dyads or triads). Thus, we use separate co-morbidity estimates to determine overlap. Reference about chronic conditions (14) gives US estimates for the proportion of the adult population with 0, 1 or 2+ chronic conditions, per age group. Using this and the 500 cities data we can estimate the proportion of the population p_{HR} in each age group in each city with at least one chronic condition listed in the CDC 500 cities data (Table S4) putting them at high-risk for flu complications.

HIV: We use the data from table 20a in CDC HIV surveillance report (15) to estimate the population in each risk group living with HIV in the US (last column, 2015 data). Assuming independence between HIV and other chronic conditions, we increase the proportion of the population at high-risk for influenza to account for individuals with HIV but no other underlying conditions.

Morbid obesity: A BMI over 40kg/m^2 indicates morbid obesity, and is considered high risk for influenza. The 500 Cities Project reports the prevalence of obese people in each city with BMI over 30kg/m^2 (not necessarily morbid obesity). We use the data from table 1 in Sturm and Hattori (16) to estimate the proportion of people with BMI>30 that actually have BMI>40 (across the US); we then apply this to the 500 Cities obesity data to estimate the proportion of people who are morbidly obese in each city. Table 1 of Morgan et al. (17) suggests that 51.2% of morbidly obese adults have at least one other high risk chronic condition, and update our high-risk population estimates accordingly to account for overlap.

Pregnancy: We separately estimate the number of pregnant women in each age group and each city, following the methodology in CDC reproductive health report (18). We assume independence between any of the high-risk factors and pregnancy, and further assume that half the population are women.

Estimating high-risk proportions for children

Since the 500 Cities Project only reports data for adults 18 years and older, we take a different approach to estimating the proportion of children at high risk for severe influenza. The two most prevalent risk factors for children are asthma and obesity; we also account for childhood diabetes, HIV and cancer. From Miller et al. (19), we obtain national estimates of chronic conditions in children. For asthma, we assume that variation among cities will be similar for children and adults. Thus, we use the relative prevalences of asthma in adults to scale our estimates for children in each city. The prevalence of HIV and cancer in children are taken from CDC HIV surveillance report (15) and cancer research report (20), respectively.

We first estimate the proportion of children having either asthma, diabetes, cancer or HIV (assuming no overlap in these conditions). We estimate city-level morbid obesity in children using the estimated morbid obesity in adults multiplied by a national constant ratio for each age group estimated from Hales et al. (21), this ratio represents the prevalence in morbid obesity in children given the one observed in adults. From Morgan et al. (17), we estimate that 25% of morbidly obese children have another high-risk condition and adjust our final estimates accordingly.

Result validation

We compare our estimates for the Austin-Round Rock Metropolitan Area to published national-level estimates (22) of the proportion of each age group with underlying high risk conditions (Table A6). The biggest difference is observed in older adults, with Austin having a lower

proportion at risk for complications for COVID-19 than the national average; for 25-39 year olds the high risk proportion is slightly higher than the national average (Table S5).

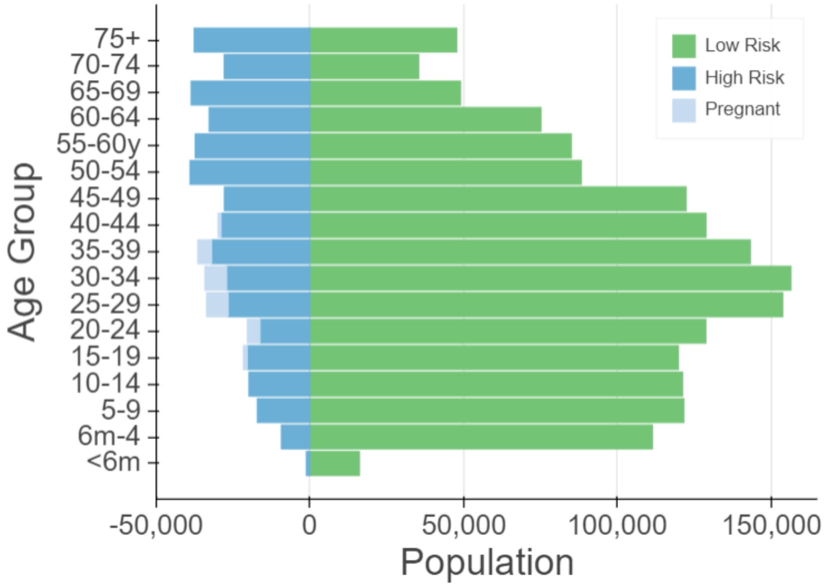

Figure S2. Demographic and risk composition of the Austin-Round Rock MSA. Bars indicate age-specific population sizes, separated by low risk, high risk, and pregnant. High risk is defined as individuals with cancer, chronic kidney disease, COPD, heart disease, stroke, asthma, diabetes, HIV/AIDS, and morbid obesity, as estimated from the CDC 500 Cities Project (12), reported HIV prevalence (15) and reported morbid obesity prevalence (16, 17), corrected for multiple conditions. The population of pregnant women is derived using the CDC's method combining fertility, abortion and fetal loss rates (23–25).

Table S4. High-risk conditions for influenza and data sources for prevalence estimation

Condition	Data source
Cancer (except skin), chronic kidney disease, COPD, coronary heart disease, stroke, asthma, diabetes	CDC 500 cities (12)
HIV/AIDS	CDC HIV Surveillance report (15)
Obesity	CDC 500 cities (12), Sturm and Hattori (16), Morgan et al. (17)
Pregnancy	National Vital Statistics Reports (23) and abortion data (24)

Table S5. Comparison between published national estimates and Austin-Round Rock MSA estimates of the percent of the population at high-risk of influenza/COVID-19 complications.

**Age Group**	**National estimates** (21)	**Austin-Round Rock (excluding pregnancy)**	**Pregnant women (proportion of age group)**
0 to 6 months	NA	6.8	-
6 months to 4 years	6.8	7.4	-
5 to 9 years	11.7	11.6	-
10 to 14 years	11.7	13.0	-
15 to 19 years	11.8	13.3	1.7
20 to 24 years	12.4	10.3	5.1
25 to 34 years	15.7	13.5	7.8
35 to 39 years	15.7	17.0	5.1
40 to 44 years	15.7	17.4	1.2
45 to 49 years	15.7	17.7	-
50 to 54 years	30.6	29.6	-
55 to 60 years	30.6	29.5	-
60 to 64 years	30.6	29.3	-
65 to 69 years	47.0	42.2	-
70 to 74 years	47.0	42.2	-
75 years and older	47.0	42.2	-

Table S6. Age and risk group population estimates from 2017 ACS survey for the Austin-Round Rock MSA (total population of 2,168,316) (26)

Age Group	Low risk	High risk
**<5**	128,527	9,350
**5-17**	327,148	37,451
**18-49**	915,894	156,209
**50-65**	249,273	108,196
**65+**	132,505	103,763

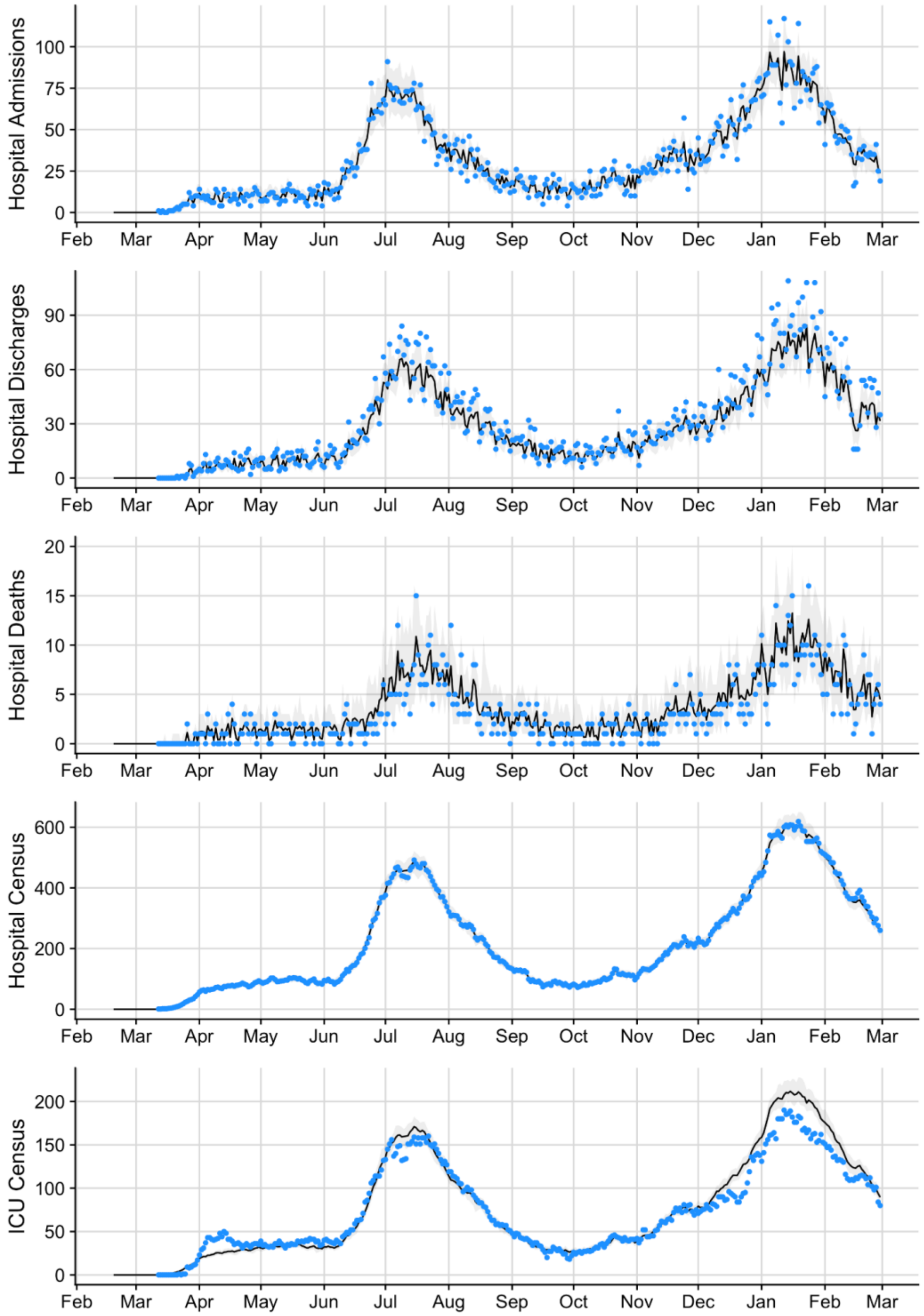

Figure S3: Comparison between data and fitted estimates from model. Model is fit to hospital admission, discharge, death, and census data. Model is not fit to daily ICU usage data, but we assume that ICU usage is 35% of hospital usage, which has been fairly stable through time. Blue dots indicate the daily data counts and the black line and shaded regions indicate the median and 95% credible interval estimates respectively.

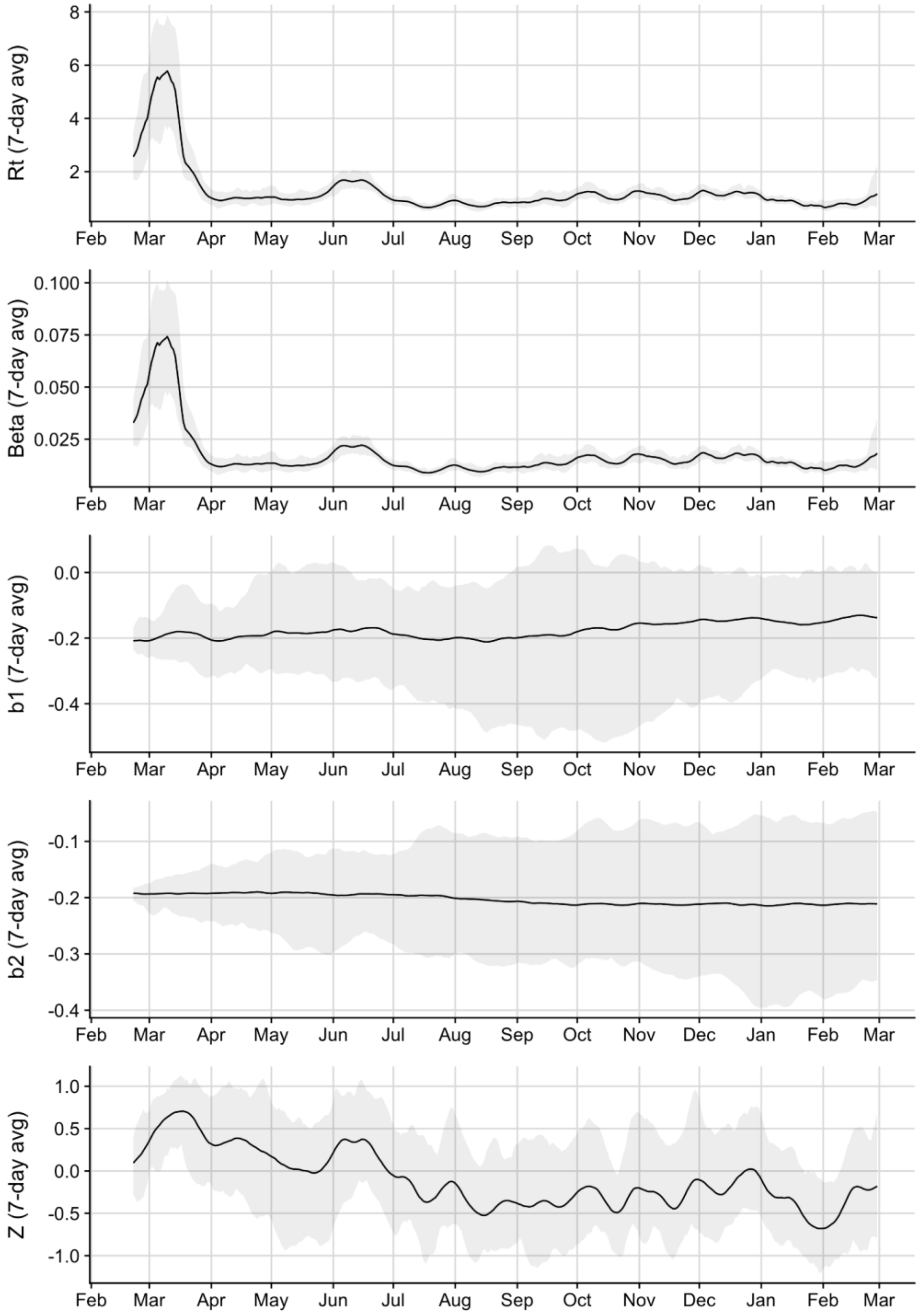

Figure S4: Fitted estimates for key parameters in the model. Black line and shaded regions indicate the median and 95% credible interval estimates respectively.

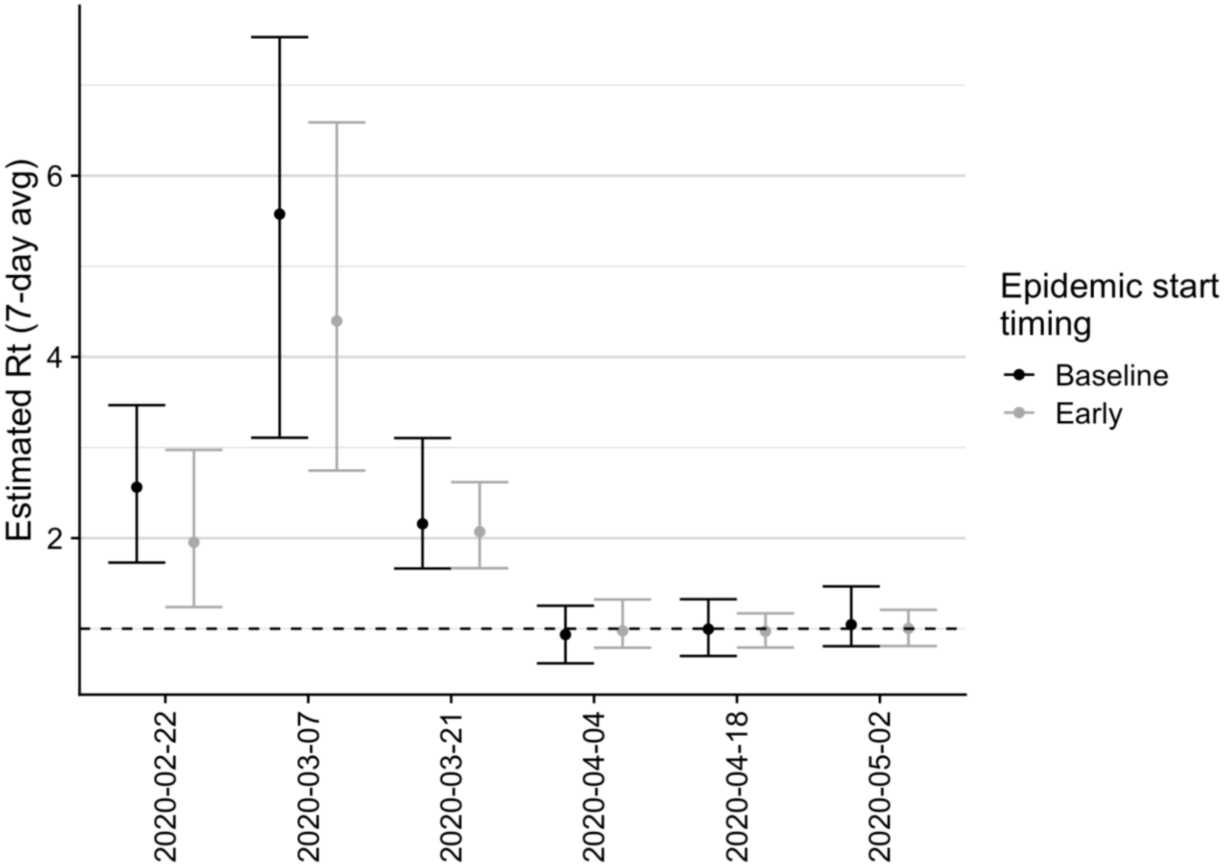

Figure S5: Estimates of the reproduction number (7-day average) for models that assume our estimated start date of February 19, 2020 compared with estimates from a model where emergence begins a month earlier on January 20, 2020. Estimates are noisiest and most uncertain during the period of time before healthcare data become available on March 13, 2020.

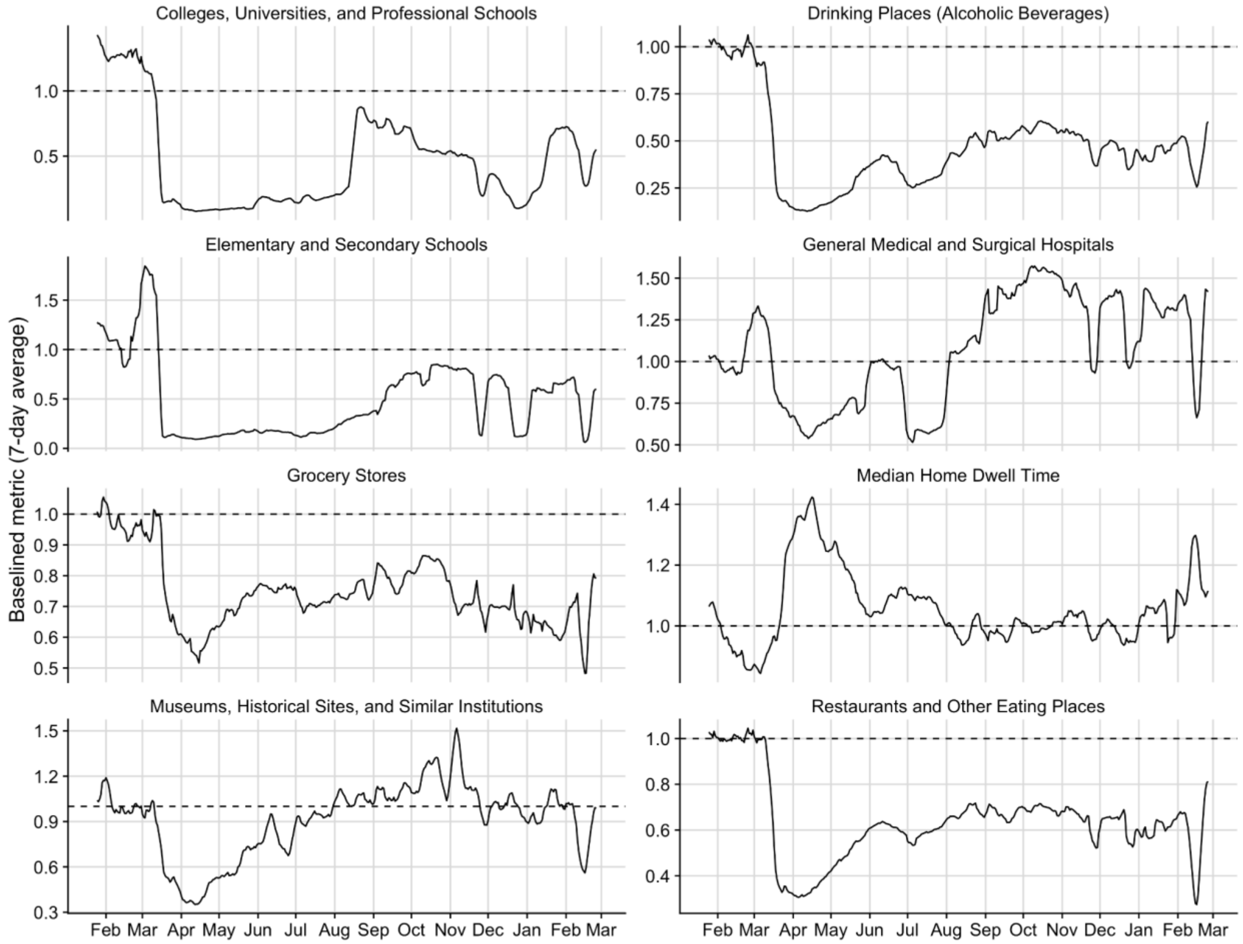

Figure S6: Seven day average for individual mobility components that feed PCA in model. Each metric is normalized to one where a value of one indicates similar mobility levels compared to the average from January and February 2020.

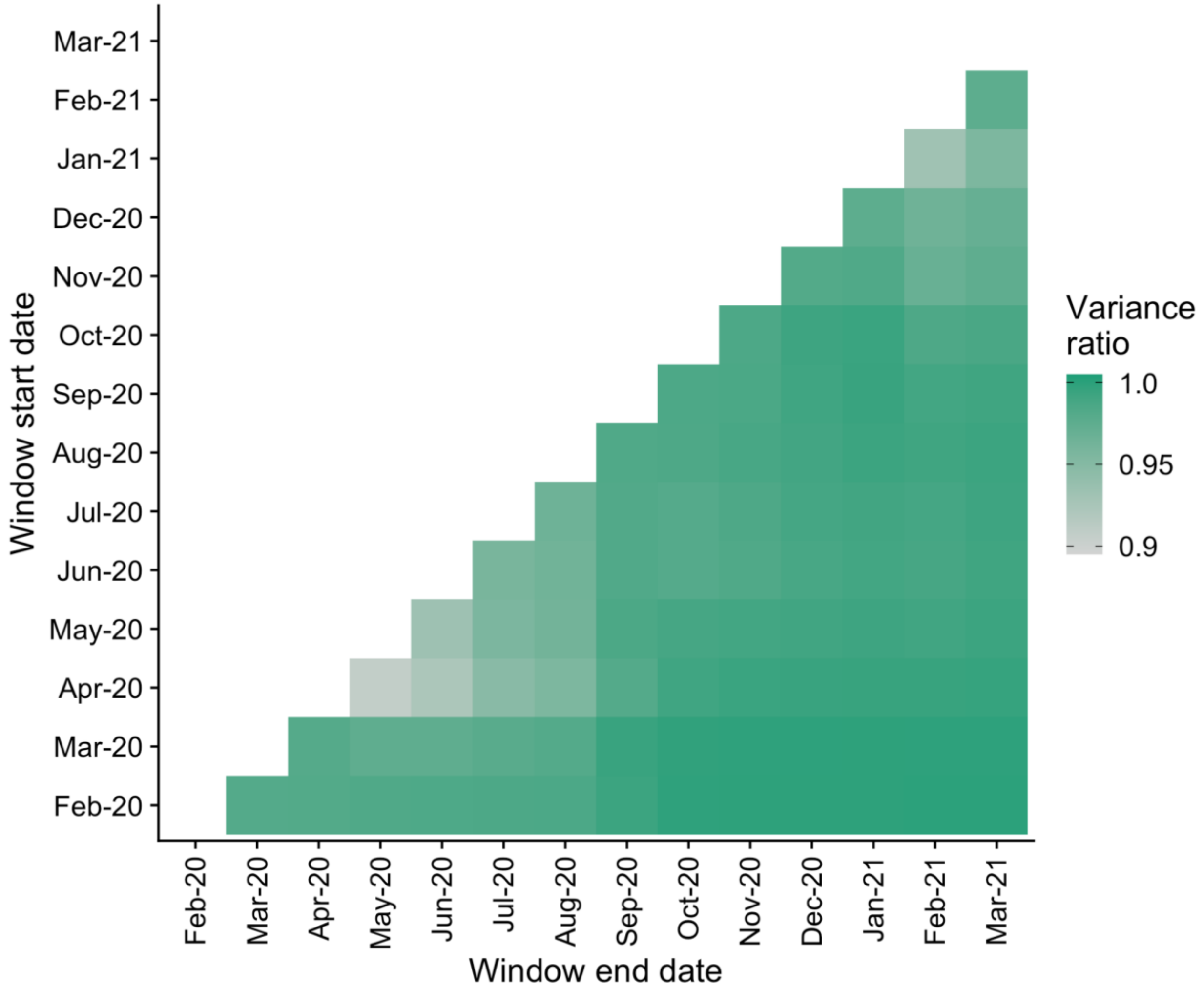

Figure S7: Sensitivity analysis for principal component analysis of mobility data. We compare the variance explained in the first two principal components from the full time window used in the model (from February 2020 to March 2020) against principal components calculated across all potential time windows indicated by a start date on the Y-axis and end date on the X-axis. A variance ratio of 1.0 indicates that 100% of the variation in the smaller window is captured by the principal components from the larger window. Overall, the full time window captures more than 90% variation across all potential sliding windows.

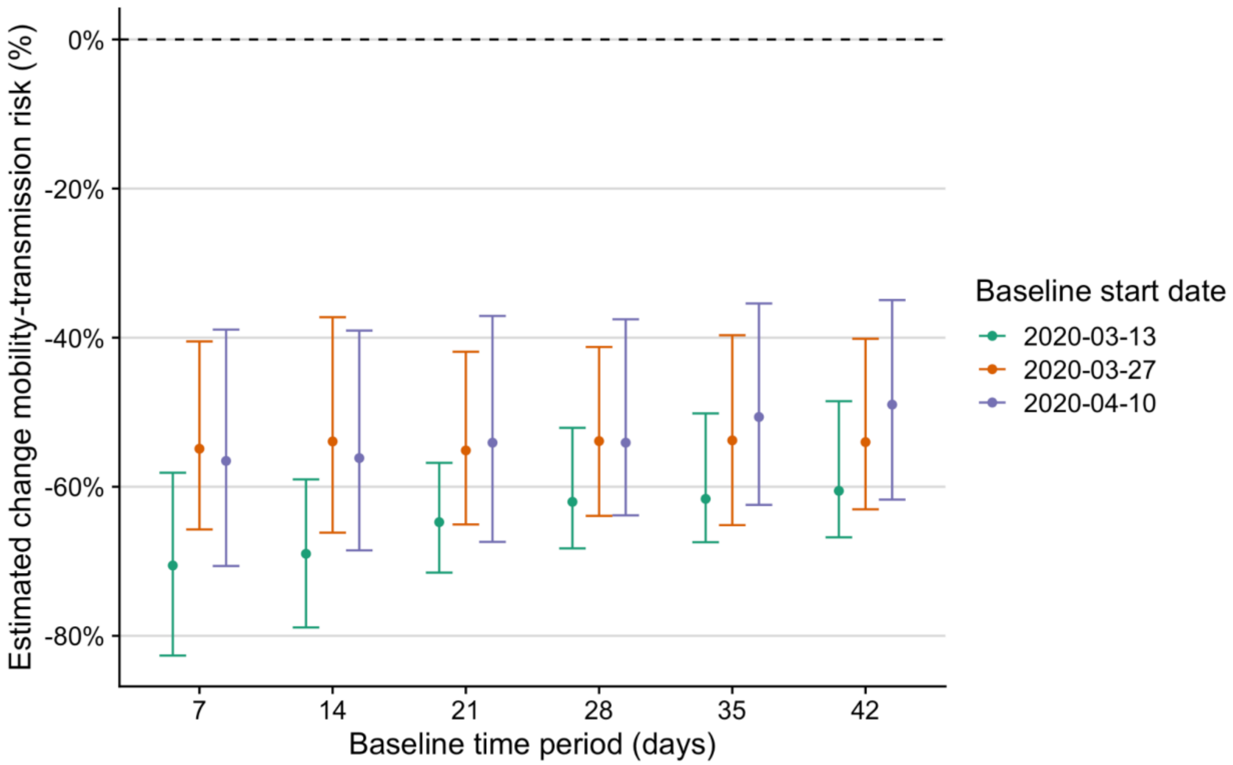

Figure S8: Sensitivity analysis for the estimated reduction in mobility-transmission risk on February 14, 2021 with respect to the start date (colors) and duration (x-axis) of the assumed baseline time period. A change of 0% means that a given amount of mobility produced the same level of transmission on February 14, 2021 as during the baseline period, while negative changes mean that a given amount of mobility corresponded to low levels of transmission on February 14, 2021 than during the baseline period. The manuscripts provides results based on a four-week baseline period (28 day) starting March 13, 2020.

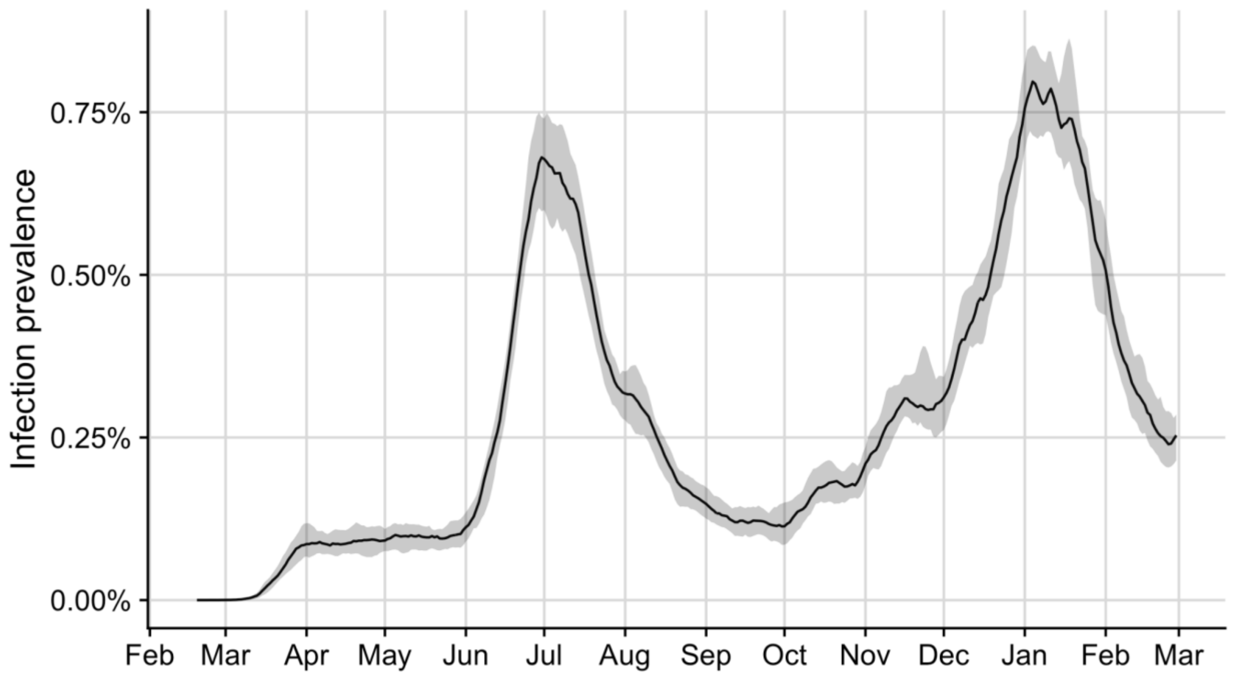

Figure S9: Estimated prevalence of symptomatic and asymptomatic SARS-CoV-2 infections, with gray 95% credible band.

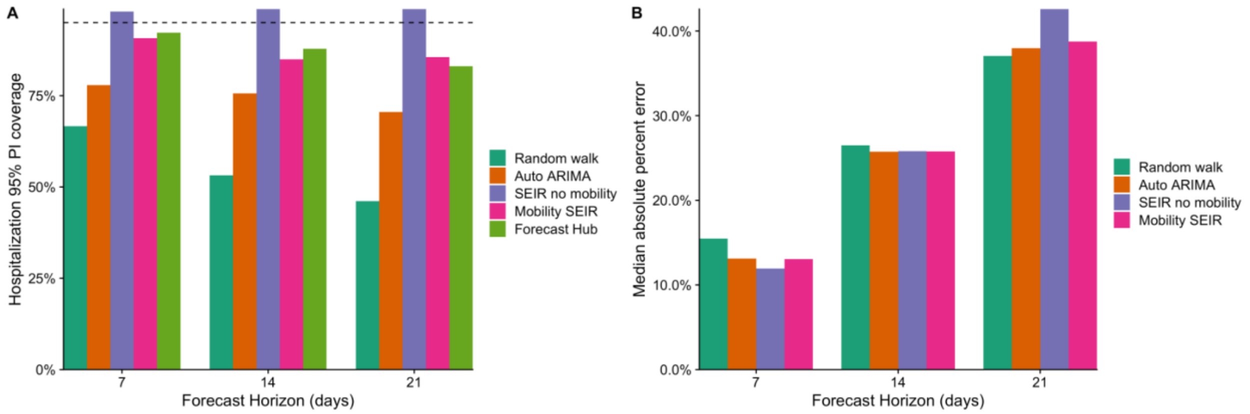

Figure S10: Comparing forecast prediction interval coverage and accuracy from April 1, 2020 to February 1, 2021. (A) Projection coverage across the four forecasting models and compared to Forecast hub COVID-19 healthcare projections (27). Coverage is estimated as the proportion of data points that fall within the 95% prediction intervals of the models one, two, and three week projections. Horizontal dashed line indicates the ideal coverage for a 95% prediction interval. (B) Forecasting accuracy as estimated by the median absolute percent error for one, two, and three week projections across all models. Higher values indicate more error.

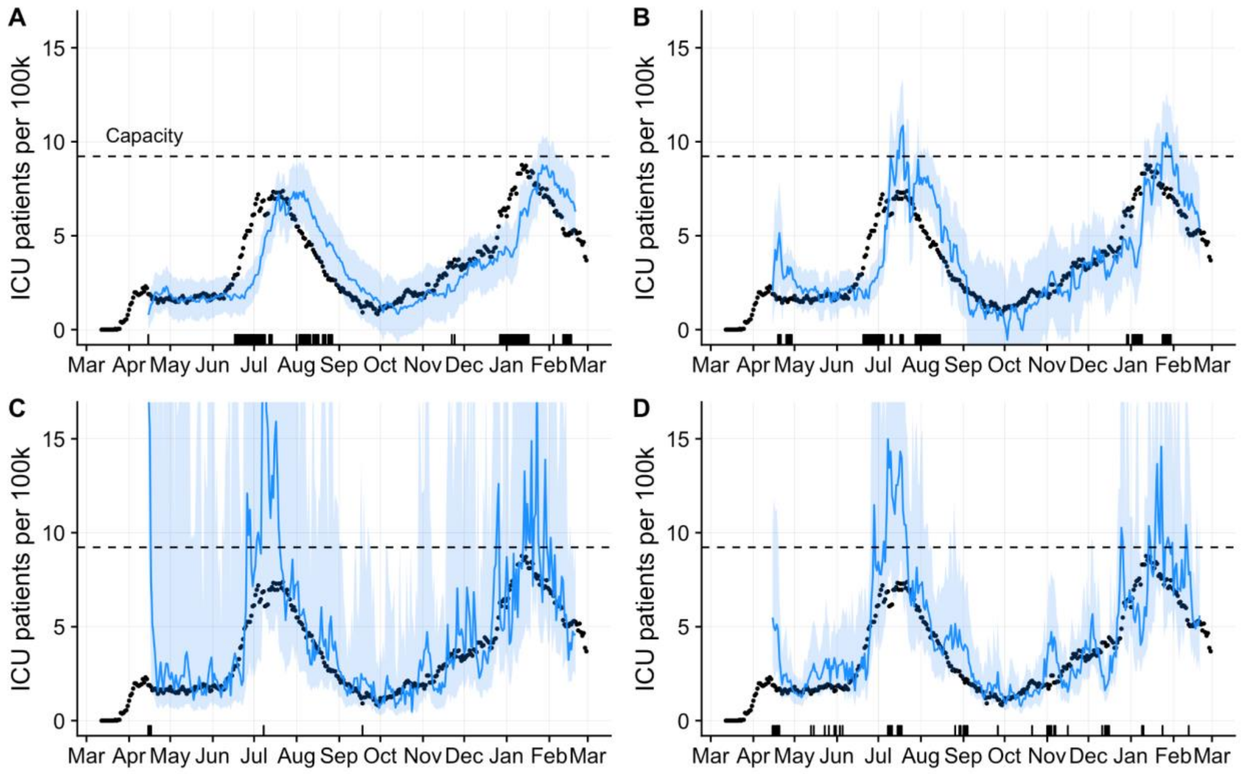

Figure S11: Comparison of two-week-ahead COVID-19 ICU projections for four models, from April 1, 2020 through February 1, 2021. Observed data (black points) are superimposed on forecasts using (A) a random walk model, (B) an auto-generated ARIMA model, (C) a simplified version of our model omitting the mobility covariate, and (D) the full version of our model. Blue lines and shading represent medians and 95% prediction intervals, respectively, across 1,000 stochastic projections. The tick marks on the x-axis indicate days on which the observed ICU usage fell outside of the one-week-ahead 95% prediction interval. The horizontal dashed line indicates the estimated ICU capacity of 200 beds for the Austin metropolitan area.

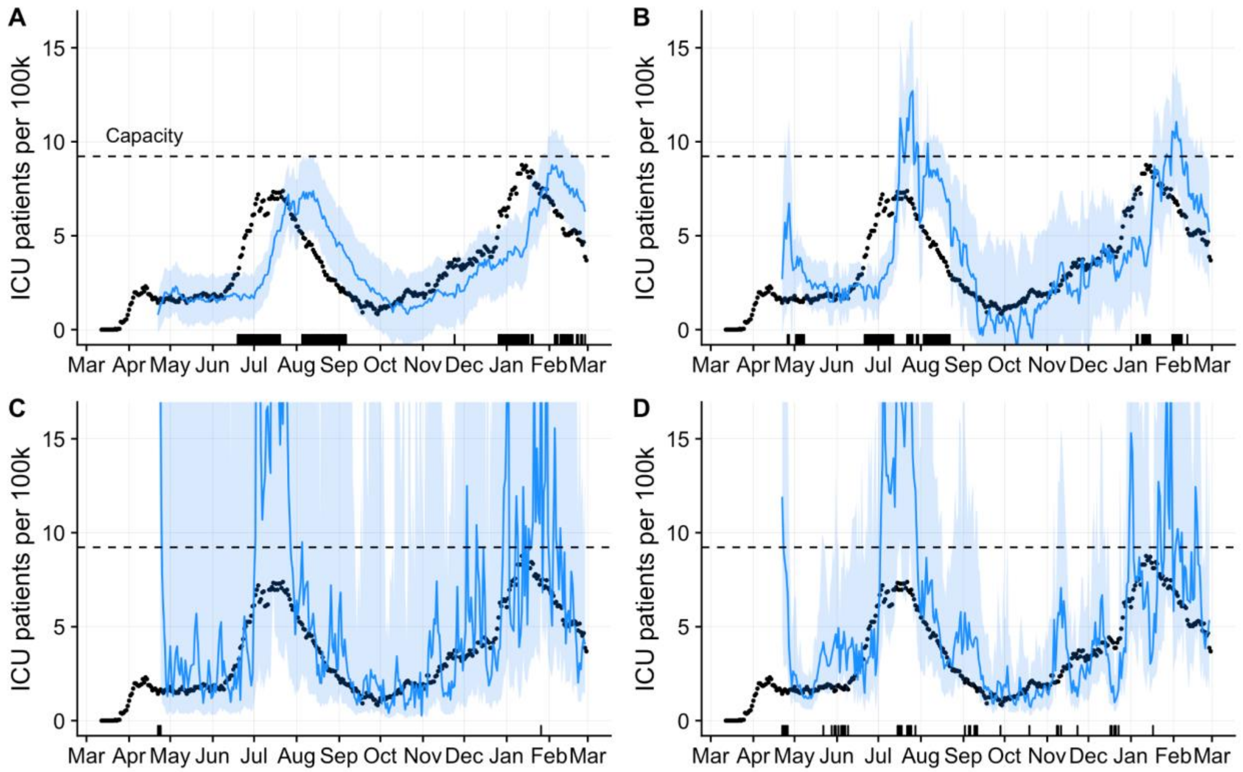

Figure S12: Comparison of three-week-ahead COVID-19 ICU projections for four models, from April 1, 2020 through February 1, 2021. Observed data (black points) are superimposed on forecasts using (A) a random walk model, (B) an auto-generated ARIMA model, (C) a simplified version of our model omitting the mobility covariate, and (D) the full version of our model. Blue lines and shading represent medians and 95% prediction intervals, respectively, across 1,000 stochastic projections. The tick marks on the x-axis indicate days on which the observed ICU usage fell outside of the one-week-ahead 95% prediction interval. The horizontal dashed line indicates the estimated ICU capacity of 200 beds for the Austin metropolitan area.

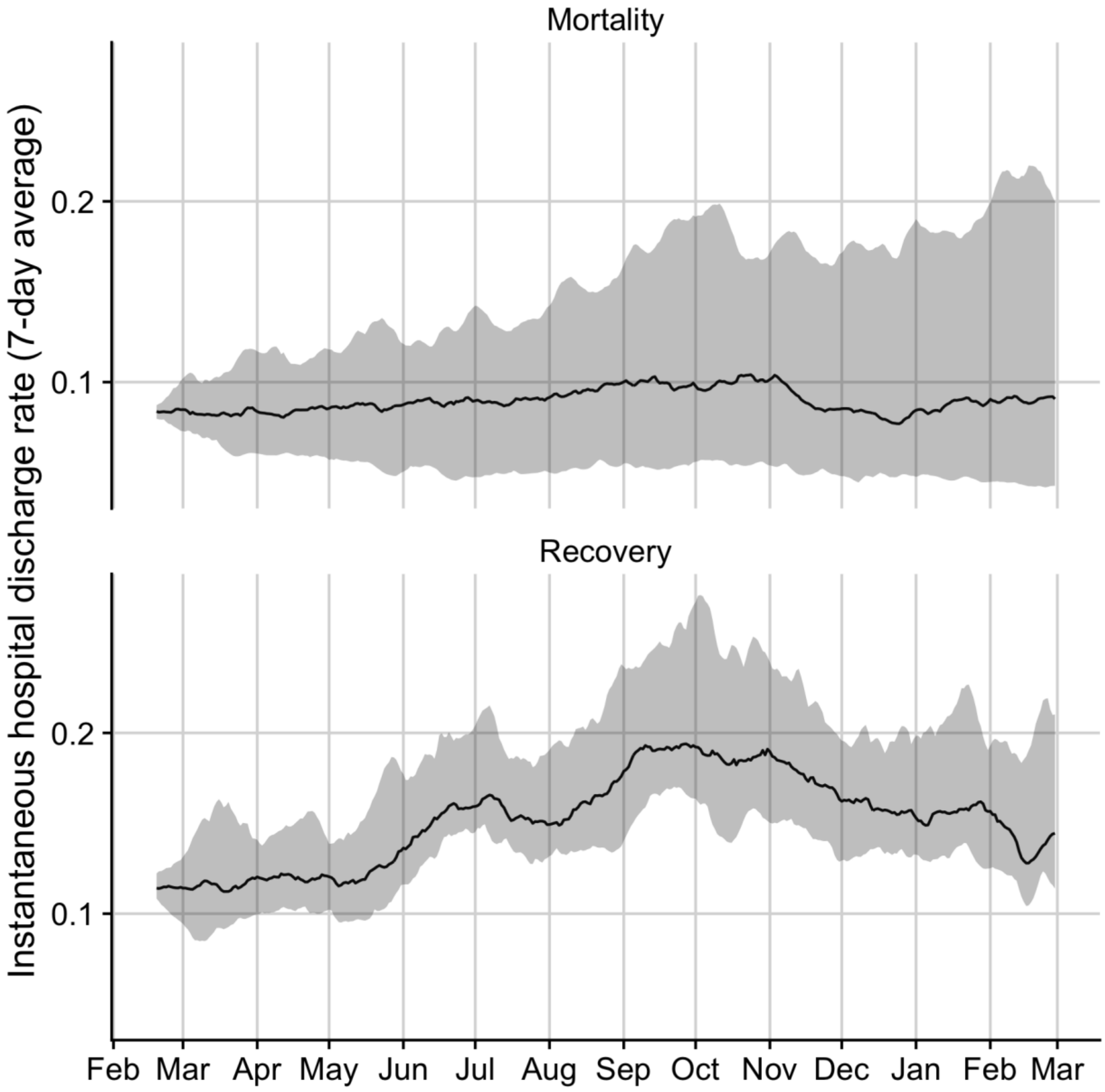

Figure S13: Estimated instantaneous rate of leaving the hospital compartment for patients discharged alive or deceased from February 18, 2020 to February 28, 2021. Lines indicate median estimates of the 7-day average rate, and gray regions indicate the 95% credible intervals.

Table S7: Key dates and events throughout the COVID-19 pandemic in Austin

Date	Event Label	Jurisdiction	Description	Citation
2020-03-13	First case	Austin	First reported case confirmed in the region	(28)
2020-03-13	School closed	Austin	Austin Independence School District and the University of Texas at Austin close for the day	(28)
2020-03-24	SH-WS	Austin	Closure of all non-essential businesses, essential movement only, and restrictions on gathering with non-household individuals	(29)
2020-05-01	Phase 1 reopening	Texas	Reopening of retail stores, restaurants, movie theaters, malls, museums, and libraries at 25-50% capacity depending on local conditions. Initiation of health safety protocols at these locations.	(30)
2020-05-18	Phase 2 reopening	Texas	Reopening of all businesses including child care centers, bars, offices, and non-essential manufacturing at 25% capacity	(31)
2020-06-15	Local mask order	Austin	Corresponds to Austin moving into “Stage 4”. All persons over the age of six required to wear a form of face covering. Also limits gathering sizes.	(32)
2020-06-26	Bars closed	Texas	All bars and similar establishments closed to in-person dining, rafting and tubing businesses closed, and outdoor gatherings limited to 100 people unless approved.	(33)
2020-07-03	State mask order	Texas	Requires face coverings in public for counties with more than 20 COVID-19 positive cases.	(34)
2020-08-26	UT reopens	Austin	The University of Texas at Austin begins Fall semester with 22,000 students expected to arrive in the City of Austin with almost all classes offered online.	(35, 36)
2020-10-05	AISD reopens	Austin	Austin Independent School District begins in-person classes at 25% capacity, phasing to 50% capacity over four weeks.	(37)
2020-10-14	Bar reopening	Texas	Bars in regions with low COVID-19 hospitalizations are allowed to open at 50% capacity, though County Judges can choose whether to opt-in. Increases capacity at other businesses to 75%	(38)
2020-12-23	Austin stage 5	Austin	No gatherings with anyone outside of the household, advised to avoid all non-essential travel, businesses suggested to operate contactless.	(39)
2021-01-09	ACS activated	Austin	Austin activates an Alternate Care Site (ACS) at the Austin Convention Center to expand healthcare capacity in the region.	(40)
2021-01-10	GA 32 enacted	Texas	Reduces business capacity from 75% to 50%, and stops hospitals from performing elective surgeries.	(41)
2021-01-30	GA 32 lifted	Texas	Removes statewide restrictions	(42)

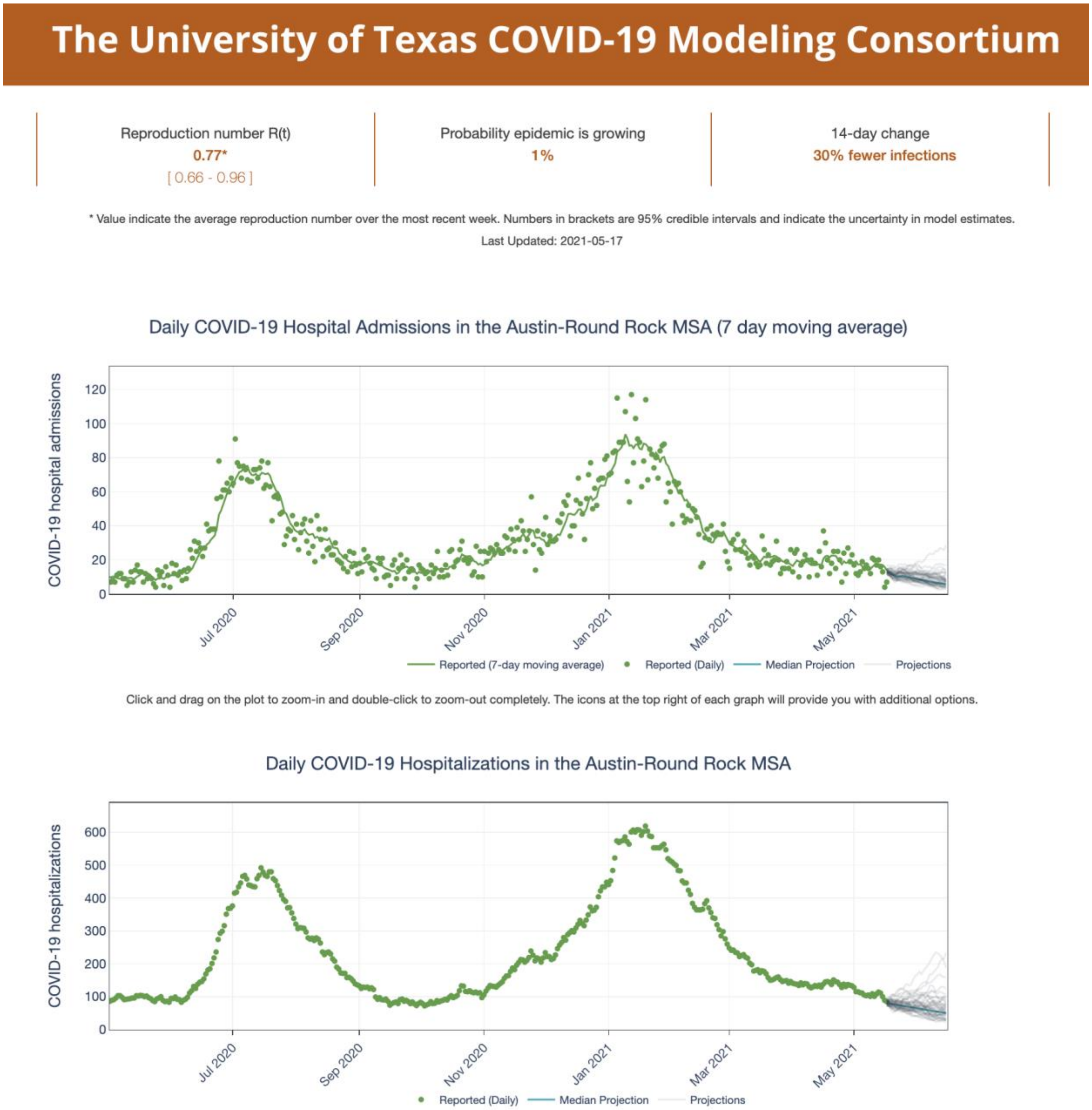

Figure S14: Top half of the Austin COVID-19 Healthcare Projections Dashboard (https://covid-19.tacc.utexas.edu/dashboards/austin/).

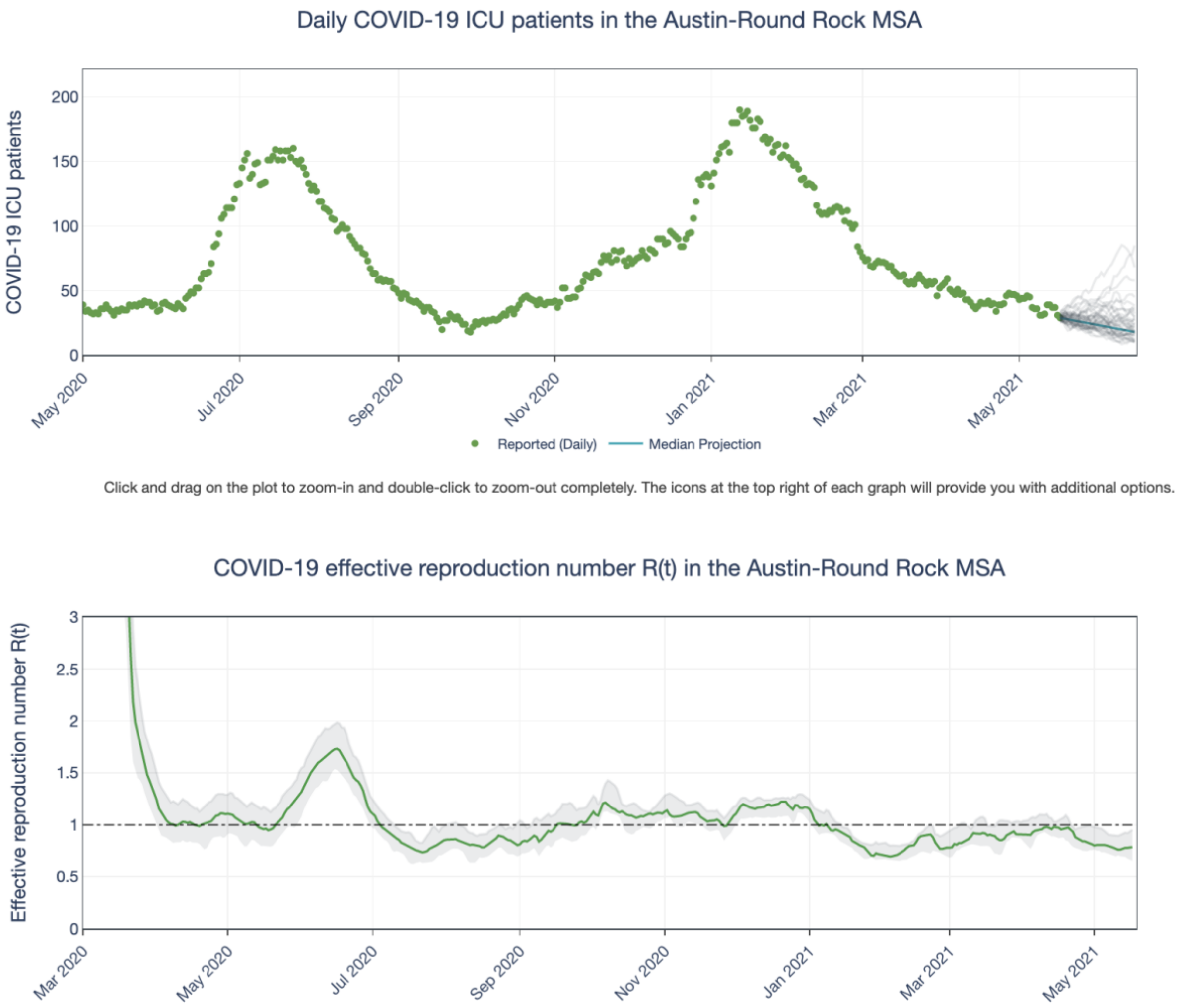

Figure S15: Bottom half of the Austin COVID-19 Healthcare Projections Dashboard (https://covid-19.tacc.utexas.edu/dashboards/austin/).

References

1. X. He, *et al.*, Temporal dynamics in viral shedding and transmissibility of COVID-19. *Nat. Med.* (2020) https:/doi.org/10.1038/s41591-020-0869-5.

2. D. F. Gudbjartsson, *et al.*, Spread of SARS-CoV-2 in the Icelandic Population. *N. Engl. J. Med.* **382**, 2302–2315 (2020).

3. J. Zhang, *et al.*, Evolving epidemiology and transmission dynamics of coronavirus disease 2019 outside Hubei province, China: a descriptive and modelling study. *Lancet Infect. Dis.* (2020) https:/doi.org/10.1016/S1473-3099(20)30230-9.

4. D. He, *et al.*, The relative transmissibility of asymptomatic COVID-19 infections among close contacts. *Int. J. Infect. Dis.* **94**, 145–147 (2020).

5. R. Verity, *et al.*, Estimates of the severity of COVID-19 disease. *medRxiv* (2020) https:/doi.org/10.1101/2020.03.09.20033357.

6. CDC, People at High Risk of Flu. *Centers for Disease Control and Prevention* (2019) (March 26, 2020).

7. CDC - BRFSS. Available at https://www.cdc.gov/brfss/index.html (2019) (March 26, 2020).

8. X. Zhang, *et al.*, Multilevel regression and poststratification for small-area estimation of population health outcomes: a case study of chronic obstructive pulmonary disease prevalence using the behavioral risk factor surveillance system. *Am. J. Epidemiol.* **179**, 1025–1033 (2014).

9. L. Tindale, *et al.*, Transmission interval estimates suggest pre-symptomatic spread of COVID-19. *medRxiv* (2020) https:/doi.org/10.1101/2020.03.03.20029983.

10. Y. Liu, A. A. Gayle, A. Wilder-Smith, J. Rocklöv, The reproductive number of COVID-19 is higher compared to SARS coronavirus. *J. Travel Med.* **27** (2020).

11. J. T. Davis, *et al.*, Cryptic transmission of SARS-CoV-2 and the first COVID-19 wave. *Nature* (2021) https:/doi.org/10.1038/s41586-021-04130-w.

12. 500 Cities Project: Local data for better health | Home page | CDC. Available at https://www.cdc.gov/500cities/index.htm (2019) (March 19, 2020).

13. Health Outcomes | 500 Cities. Available at https://www.cdc.gov/500cities/definitions/health-outcomes.htm (2019) (March 28, 2020).

14. Part One: Who Lives with Chronic Conditions. *Pew Research Center: Internet, Science & Tech* (2013) (November 23, 2019).

15. C. for Disease Control, Prevention, Others, HIV surveillance report. 2016; 28. *URL: http://www. cdc. gov/hiv/library/reports/hiv-surveil-lance. html. Published November* (2017).

16. R. Sturm, A. Hattori, Morbid obesity rates continue to rise rapidly in the United States. *Int. J. Obes.* **37**, 889–891 (2013).

17. O. W. Morgan, *et al.*, Morbid obesity as a risk factor for hospitalization and death due to 2009 pandemic influenza A(H1N1) disease. *PLoS One* **5**, e9694 (2010).

18. "Estimating the Number of Pregnant Women in a Geographic Area from CDC Division of Reproductive Health. Available at https://www.cdc.gov/reproductivehealth/emergency/pdfs/PregnacyEstimatoBrochure508.pdf."

19. G. F. Miller, E. Coffield, Z. Leroy, R. Wallin, Prevalence and Costs of Five Chronic Conditions in Children. *J. Sch. Nurs.* **32**, 357–364 (2016).

20. Cancer Facts & Figures 2014. Available at https://www.cancer.org/research/cancer-facts-statistics/all-cancer-facts-figures/cancer-facts-figures-2014.html (March 30, 2020).

21. C. M. Hales, C. D. Fryar, M. D. Carroll, D. S. Freedman, C. L. Ogden, Trends in Obesity and Severe Obesity Prevalence in US Youth and Adults by Sex and Age, 2007-2008 to 2015-2016. *JAMA* **319**, 1723–1725 (2018).

22. R. K. Zimmerman, D. S. Lauderdale, S. M. Tan, D. K. Wagener, Prevalence of high-risk indications for influenza vaccine varies by age, race, and income. *Vaccine* **28**, 6470–6477 (2010).

23. J. A. Martin, B. E. Hamilton, M. J. K. Osterman, A. K. Driscoll, P. Drake, Births: Final Data for 2017. *Natl. Vital Stat. Rep.* **67**, 1–50 (2018).

24. T. C. Jatlaoui, *et al.*, Abortion Surveillance - United States, 2015. *MMWR Surveill. Summ.* **67**, 1–45 (2018).

25. S. J. Ventura, S. C. Curtin, J. C. Abma, S. K. Henshaw, Estimated pregnancy rates and rates of pregnancy outcomes for the United States, 1990-2008. *Natl. Vital Stat. Rep.* **60**, 1–21 (2012).

26. US Census Bureau, American Community Survey (ACS) (January 18, 2021).

27. Reich, Nicholas G. , Tibshirani, Ryan J., Ray, Evan L., Rosenfeld, Roni, On the predictability of COVID-19 (2021) (October 21, 2021).

28. R. Mack, Austin health officials say three people being treated for COVID-19; UT and Austin ISD schools close for the day. *The Texas Tribune* (2020) (April 15, 2021).

29. Mayor Steve Adler, Austin STAY HOME - WORK SAFE Order. Available at http://www.austintexas.gov/edims/document.cfm?id=337824 (2020).

30. Governor Abbott announces phase one to open Texas, establishes statewide minimum standard health protocols. Available at https://gov.texas.gov/news/post/governor-abbott-announces-phase-one-to-open-texas-establishes-statewide-minimum-standard-health-protocols (April 16, 2021).

31. Governor Abbott announces phase two to open Texas. Available at https://gov.texas.gov/news/post/governor-abbott-announces-phase-two-to-open-texas (April 16, 2021).

32. Travis County enters into Stage 4. Available at https://www.austintexas.gov/news/austin-

travis-county-enters-stage-4 (February 23, 2021).

33. Governor Abbott takes executive action to contain spread of COVID-19. Available at https://gov.texas.gov/news/post/governor-abbott-takes-executive-action-to-contain-spread-of-covid-19 (February 3, 2021).

34. Governor Abbott establishes statewide face covering requirement, issues proclamation to limit gatherings. Available at https://gov.texas.gov/news/post/governor-abbott-establishes-statewide-face-covering-requirement-issues-proclamation-to-limit-gatherings (April 19, 2021).

35. J. Hartzell, UT Austin Fall 2020 Reopening Plans. Available at https://president.utexas.edu/messages-speeches-2020/fall-2020-reopening-plans (2020) (March 24, 2021).

36. COVID-19 Campus Introduction and Gathering Risks for Reopening the University of Texas at Austin. Available at https://doi.org/10.15781/aa7a-0c58 https:/doi.org/10.15781/aa7a-0c58 (April 19, 2021).

37. AISD Open for Learning Newsletter: First Week, Afterschool Care and More. Available at https://www.austinisd.org/announcements/2020/10/09/aisd-open-learning-newsletter-first-week-afterschool-care-and-more-boletin (April 19, 2021).

38. Governor Abbott issues executive order to open bars in qualifying counties. Available at https://gov.texas.gov/news/post/governor-abbott-issues-executive-order-to-open-bars-in-qualifying-counties (April 1, 2021).

39. Austin Public Health increases COVID-19 risk to stage 5, the highest level. Available at https://www.austintexas.gov/news/austin-public-health-increases-covid-19-risk-stage-5-highest-level (April 5, 2021).

40. Austin, Travis County begins work to open alternate care site. Available at https://www.austintexas.gov/news/austin-travis-county-begins-work-open-alternate-care-site (April 5, 2021).

41. Governor's order GA 32 activated for trauma service area that includes Austin-Travis county. Available at https://www.austintexas.gov/news/governors-order-ga-32-activated-trauma-service-area-includes-austin-travis-county (April 5, 2021).

42. Governor's Order GA 32 Restrictions Cancelled for Trauma Service Area that Includes Austin-Travis County. Available at https://austintexas.gov/news/governors-order-ga-32-restrictions-cancelled-trauma-service-area-includes-austin-travis-county (May 7, 2021).
